# Nucleophosmin Protein Dephosphorylation by DUSP3 Is a Fine-Tuning Regulator of p53 Signaling to Maintain Genomic Stability

**DOI:** 10.3389/fcell.2021.624933

**Published:** 2021-03-11

**Authors:** Lilian C. Russo, Pault Y. M. Ferruzo, Fabio L. Forti

**Affiliations:** Laboratory of Biomolecular Systems Signalling, Department of Biochemistry, Institute of Chemistry, University of São Paulo, São Paulo, Brazil

**Keywords:** nucleophosmin (NPM), DUSP3/VHR, p53, genomic stability, tyrosine dephosphorylation, NPM translocation

## Abstract

The dual-specificity phosphatase 3 (DUSP3), an atypical protein tyrosine phosphatase (PTP), regulates cell cycle checkpoints and DNA repair pathways under conditions of genotoxic stress. DUSP3 interacts with the nucleophosmin protein (NPM) in the cell nucleus after UV-radiation, implying a potential role for this interaction in mechanisms of genomic stability. Here, we show a high-affinity binding between DUSP3-NPM and NPM tyrosine phosphorylation after UV stress, which is increased in DUSP3 knockdown cells. Specific antibodies designed to the four phosphorylated NPM’s tyrosines revealed that DUSP3 dephosphorylates Y29, Y67, and Y271 after UV-radiation. DUSP3 knockdown causes early nucleolus exit of NPM and ARF proteins allowing them to disrupt the HDM2-p53 interaction in the nucleoplasm after UV-stress. The anticipated p53 release from proteasome degradation increased p53-Ser15 phosphorylation, prolonged p53 half-life, and enhanced p53 transcriptional activity. The regular dephosphorylation of NPM’s tyrosines by DUSP3 balances the p53 functioning and favors the repair of UV-promoted DNA lesions needed for the maintenance of genomic stability.

## Introduction

Nucleophosmin (NPM) is a major RNA-associated nucleolar phosphoprotein that contains a central long sequence rich in acidic amino acids with high affinity for silver ions (Schmidt-Zachmann et al., 1987; Schmidt-Zachmann and Franke, 1988). It is encoded by the *NPM* gene in a 296-aa long protein exhibiting structurally well-defined C-terminal and N-terminal ends, the latter containing the foremost domains responsible for the pentameric homo-oligomer structure (Hyung et al., 2007; Grummitt et al., 2008). NPM is associated with mRNA processing, ribosomal biogenesis, cell proliferation, and duplication of centrosomes, mainly in tumor cells (Grisendi et al., 2006). NPM phosphorylation at Thr199 plays a crucial role in the RNF8-dependent DNA repair after double breakage of the DNA strands induced by ionizing radiation (Koike et al., 2010). It was demonstrated that NPM is related to DNA repair by base excision repair (BER), by controlling levels, regulating activity, and modulating the nucleolar location of many enzymes of this pathway (Poletto et al., 2014; Vascotto et al., 2014). In addition, its overexpression in fibroblasts causes greater resistance to ultraviolet radiation, proposing that this protein is associated with the specific repair of DNA damage caused by this radiation through the nucleotide excision repair (NER) pathway (Wu, 2002). It has been shown that nucleolar NPM levels set a threshold for p53 phosphorylation in response to ultraviolet radiation, due to a possible competition between these two proteins for ATR phosphorylation (Maiguel et al., 2004) and for binding and inhibition of the MDM2 protein, a specific ubiquitin ligase that constantly mediates the degradation of p53 during cell stress (Oren, 1999). After DNA damage caused by ultraviolet or gamma radiation, NPM can also binds to chromatin and regulates the expression of p53, showing its other face and participation in the DNA repair pathway (Lee S. Y. et al., 2005). Thus, NPM seems to be a promising target in cell sensitization for chemo or radiotherapies (Sekhar et al., 2014), especially because it impacts on the functional regulation of the axis formed by p53, HDM2, and ARF protein (Kurki et al., 2004a,b; Luchinat et al., 2018).

An aspect of NPM physiology in most tumor cells that remains mostly unexplored is its posttranslational regulation, since its function, localization, and mobility within cells are highly regulated by phosphorylation events. *In silico* prediction and biochemical *in vitro* studies have shown that NPM protein structure comprises unexplored phosphosites that could potentially regulate its functions, particularly on phosphotyrosine residues (Ramos-Echazábal et al., 2012). NPM physically interacts and colocalizes with the DUSP3 protein in cells preferentially under conditions of genomic instability (Panico and Forti, 2013). DUSP3 or VHR (vaccinia virus H1-related phosphatase) is a dual-specificity phosphatase (DSP or DUSP) belonging to the class I PTP that dephosphorylates Tyr and Thr residues, and was the first DUSP identified in mammals (Ishibashi et al., 1992). It is widely expressed and active in cells from several tissues, where it is preferentially found in the nucleus. Differently from other DUSPs, this enzyme expression is not regulated in response to extracellular stimuli that activate the mitogen-activated protein kinases (MAPK) (Kondoh and Nishida, 2007). DUSP3 can dephosphorylate ERK1/2 (Alonso et al., 2001), STAT5 (Hoyt et al., 2007), ERBB2 (Wang et al., 2011), FAK (Chen et al., 2017), STAT3 (Kim et al., 2020), and possibly other substrates. By acting upon these substrates, DUSP3 was demonstrated to mediate or regulate many cellular processes, such as cell cycle arrest and senescence, apoptosis, cell adhesion, migration, and metastasis (Russo et al., 2018).

By contrast to protein serine/threonine phosphatases (PSPs), which have been explored since a decade before, PTPs are increasingly emerging as important regulators of genomic stability, and in particular the DUSP3, which is a versatile enzyme with potentialities as a therapeutic target due to its protein partners identified in conditions of genotoxic stress (Forti, 2015; Monteiro et al., 2019). In addition to that, the loss of DUSP3 was shown to negatively interfere with the functioning of the DDR, HR, NHEJ, and NER pathways (Torres et al., 2017; Russo et al., 2020), although by molecular mechanisms are not yet understood. Therefore, here we focused on the molecular mechanisms used by DUSP3-NPM interaction to affect the abovementioned cellular responses and found out that DUSP3 dephosphorylates three tyrosine residues (Y29, Y67, and Y271) of NPM. These dephosphorylations affect the homo-oligomerization equilibrium of NPM, its nucleolus-nucleoplasm translocation rate, as well as the subnuclear (re)localization of ARF and HDM2, which collectively culminate in increased stability, phosphorylation, and transcriptional activity of the p53 protein under conditions of genotoxic stress.

## Materials and Methods

### Cell Culture and Treatments

MRC-5V1 (MRC-5) and XP12RO (XPA) cell lines (Huschtscha and Holliday, 1983; Satokata et al., 1992) were cultured in Dulbecco’s modified Eagle’s medium (DMEM) containing 10% FBS, penicillin (100 U/ml) and streptomycin (100 g/ml) (Life Technologies) under incubation at 37°C and 5% CO_2_. DUSP3 knockdown cells (named as shDUSP3) and the negative control cells containing a scramble sequence with no homology to existing mRNA (non-silencing, named as NS) were maintained in culture medium containing 0.75 μg/ml puromycin as described (Russo et al., 2020). For the UV treatments, cells were transferred to PBS to undergo irradiation using a lamp with wavelength corresponding to UVC radiation (260 nm): doses of 6, 18, or 28 J/m^2^ were used for different experiments. A VLX-3W dosimeter (Vilber Lourmat, Eberhardzell, Baden-Württemberg, Germany) was used to calibrate the UV lamp. Gamma ionizing radiation (IR) treatment (15 Gy) was carried out in a Cobalt-60 irradiator model Gamacell 220, located at the Institute of Energy and Nuclear Research (IPEN). For the cycloheximide (CHX) treatments, a 100-ng/ml stock solution in DMSO was diluted in DMEM and added to the cells at 37°C until the desired collection time for cell lyses.

### Phospho-Y-NPM Antibody Production

Phospho-Y-decapeptides were designed for each of the four tyrosines of NPM (Y17, Y29, Y67, and Y271) and evaluated according to their immunogenicity and hydrophobicity using the Epitome tool^1^. The phospho-Y-decapeptides were purchased from Chinese Peptide Company (China) and synthesized according to the following peptide sequences: p-NPM(Y17): SPLRPQN-**pY-**L; p-NPM(Y29): ADKD-**pY-**HFKVD; p-NPM(Y67): AMN-**pY-**EGSPIK; p-NPM(Y271): EAKFIN-**pY-**VKN. Polyclonal antibodies against each phospho-Y-decapeptide of NPM were purchased from the start-up Celula B (Federal University of Rio Grande do Sul, Porto Alegre-RS, Brazil). Rabbits were individually immunized with each phospho-Y-decapeptide to provide the antiserum-containing antibodies, which were then purified by immunoaffinity chromatography (IAC) and submitted to Elisa assays for titration. All four anti phospho-Y-NPM antibodies were individually tested according to their specificity through immunoprecipitation, immunoblotting, and immunofluorescence assays using the total NPM antibody as control. The ideal conditions for these assays were extensively tested and used as described below.

### Plasmids and Recombinant Protein Purification

Plasmids used in the SPR assays were pGEX-4T2 cloned with DUSP3 cDNA (WT or the C124S mutant) (Torres et al., 2017), NPM full length (donated by Prof. Mitsuru Okuwaki, University of Tsukuba, Tsukuba-Ibaraki, Japan), ERK1 (donated by Prof. Rony Seger, The Weizmann Institute of Science, Israel), and pET21a(+) cloned with cDNA of truncated N-terminal of NPM_9–122_ (donated by Se Won Suh, Addgene plasmid #23142). For dual-luciferase reporter assays, we used pGL3-p53RE (donated by Prof. Tomas Mustelin, Sanford Burnham Prebys Institute, La Jolla-CA, United States) and pRL-SV40 vectors (donated by Prof. Carlos F. M. Menck, Institute of Biomedical Sciences—University of São Paulo, São Paulo-SP, Brazil). Recombinant proteins were expressed in BL-21 (DE3) bacteria induced by 1 mmol/L IPTG for 3 h. DUSP3 (WT, C124S), and NPM full length proteins GST-tagged were purified by affinity chromatography in Glutathione-Sepharose 4B resin (GE Healthcare), and NPM_9–122_ 6x-His-tagged was purified on His-Trap^TM^ (Sigma-Aldrich, St. Louis-MO, United States), following the manufacturer protocol. The GST-tag was specifically removed by thrombin enzyme (Sigma-Aldrich, St. Louis-MO, United States) cleavage for 18 h at 18°C and purified by molecular weight exclusion filters (Millipore).

### Surface Plasmon Resonance (SPR)

The SPR technique was used to measure physical–chemical parameters and to analyze in real time the bimolecular interactions between DUSP3-WT or DUSP3-C124S mutant (DUSP3 phosphatase dead mutation in which the catalytic cysteine 124 was substituted by a serine) and ERK1, NPM full length or NPM_9–122_. In addition, we analyzed the interaction between DUSP3-WT and the four different phospho-Y-decapeptides of NPM. The experiments were carried on the Biacore T100 equipment (GE Healthcare) at Cepid-CeTICS of Instituto Butantan, São Paulo-SP, Brazil. The immobilization of DUSP3-WT or DUSP3-C124S was performed in CM5 sensorchip (GE Healthcare) following manufacturer specifications. A total of 200 RU (Resonance Units) or 3,000 RU of DUSP3 was immobilized for the protein or peptide interaction assays, respectively. To verify the physical–chemistry parameters of interaction between DUSP3 (WT or C124S) and ERK1, NPM full length or NPM_9–122_ proteins (analytes), we performed kinetic assays at 25°C with a 12.5- to 1,000-nM variation for each analyte. The interaction phase occurred for 120 s in a constant flow (10 μl/min) in HBS-EP buffer, whereas the dissociation of proteins occurred for 300 s. The sensorchip surface was regenerated after each run with 1 M glycine pH 2.0 for 60 s (10 μl/min), followed by a 30-s stabilization period in HBS-EP buffer. The peptides were tested between 5 and 40 μM, diluted in HBS-N buffer. Individual runs were performed at 25°C for 120 s, followed by the dissociation for 300 s (10 μl/min). Regeneration step was achieved with 1 M glycine pH 2.0 for 75 s (10 μl/min), followed by a second regeneration of HBS-N buffer (60 s, 15 μl/min) and stabilization of 30 s. For each protein or peptide, three independent experiments were carried in individual sensorchips, and the concentrations were injected randomly. The data are displayed in the format of sensorgrams and tables, in RU, after subtracting the values obtained from the immobilized cell from the respective white cells. The Biacore software analyzes the association rate constant (ka) and the dissociation rate constant (kd) to provide the affinity of bimolecular interaction, also chemically known as equilibrium dissociation constant (KD). The affinity constant is assumed as KD = ka/kd, expressed as molar units (M). The mass transfer tests, essential for the determination of accurate dissociation constant values, were performed for all proteins and peptides analyzed (see Supplementary Figures 1A,2B). For this, independent runs were performed by fixing a protein or peptide concentration and varying the analyte flow between 5, 15, or 75 μl/min in the association phase.

### Immunoblottings and Immunoprecipitations

Cells were lysed with RIPA buffer (50 mmol/L Tris–HCl, pH 7.2, 1% Triton X-100, 0.5% sodium deoxycholate, 0.1% SDS, 500 mmol/L NaCl, 10 mmol/L MgCl2, 1 mM Na_3_VO_4_, 1 mM NaF, 2 μg/mL leupeptin, pepstatin, aprotinin, and 1 mmol/L PMSF) (Sigma-Aldrich, St. Louis-MO, United States), and 50 μg of total protein was mixed with Laemmli sample buffer. SDS–PAGE was performed at 11% SDS–PAGE, and proteins were transferred to a nitrocellulose membrane (Merck-Millipore, Billerica, MA, United States). The membrane blocking was in 5% non-fat dry milk in TTBS buffer [25 mM Tris–HCl, pH 7.4, and 125 mM NaCl (TBS) containing 0.1% Tween 20] for 1 h at RT. Specific antibodies against different proteins were diluted in TTBS and incubated for 18 h at 4°C: DUSP3 (1:1,000, BD Biosciences), NPM (1:1,000, Sigma-Aldrich, St. Louis-MO, United States), p53 (1:1,000, Santa Cruz, Santa Cruz-CA, United States), p-p53 (1:1,000, Cell Signalling), Actin (1:1,000, Santa Cruz, Santa Cruz-CA, United States), and HDM2 (1:1,000, Santa Cruz, Santa Cruz-CA, United States). For the antibodies for each one, the phospho-Tyr of NPM was used in blocking solution (1:200) by incubating the membranes for 18 h at 4°C. Membranes were incubated with the appropriate fluorescent secondary antibodies IR Dye 680CW or 800CW (1:15,000, LICOR, Bad Homburg, Germany), the bands were visualized in the Odyssey Infrared Imaging System (LICOR, Bad Homburg, Germany), and then analyzed/quantified using Image Studio software (LICOR, Bad Homburg, Germany). For the immunoprecipitations, the total cell lysate (250 μg) was incubated with anti-NPM antibody (2.5 μg) overnight at 4°C under horizontal rotation in RIPA buffer containing only protease inhibitors. The complex was incubated with 10 μl of Protein A/G PLUS Agarose (Santa Cruz, Santa Cruz-CA, United States) for 1 h at 4°C under horizontal rotation. The supernatant was removed, and the resin was gently washed four times with 200 μl of RIPA buffer plus protease inhibitors. After elution of the resin and denaturation in sample buffer, the proteins were separated on a 12% SDS–PAGE. Immunoblottings were performed as described, first incubating with anti-phospho-Tyr antibody (1:2,000, Sigma-Aldrich, St. Louis-MO, United States) and developed with IR Dye 680CW, and then incubated with anti-NPM (1:1,000, Sigma-Aldrich, St. Louis-MO, United States) and developed with IR Dye 800CW.

### *In vitro* Dephosphorylation Assays

Total lysate (100 μg) of MRC-5 shDUSP3 cells was obtained 3 h after UVC exposure as described before for immunoblotting and immunoprecipitation assays. Purified recombinant DUSP3-WT (4 μg) protein was produced and then incubated with cell lysate, in the presence or absence of 1 mM pan-inhibitor of PTPs, Na_3_VO_4_, and allowed to react at 37°C during 30 min or 1 h under gentle rotation. Each dephosphorylation reaction performed in triplicates was individually submitted to 12% SDS–PAGE followed by immunoblottings using individual antibodies against the specific phosphorylated residues (Y29, Y67, and Y271) and compared with the expression of total NPM and Actin present in the lysates.

### Immunofluorescence and Confocal Microscopy Assays

The cells were submitted or not to 18 J/m^2^UVC radiation, and after 0, 30 min, 3 h, 6 h and 24 h, fixed (4% paraformaldehyde and 2% sucrose), permeabilized (0.5% Triton X-100, 6.84% sucrose, 3 mM MgCl_2_ in PBS for 5 min on ice) and blocked (3% BSA and 10% SFB in PBS for 30 min at RT), with two washes in PBS. The primary antibodies p-Y-NPM, NPM, p-p53, p53, ARF, HDM2, and NAT10 (Supplementary Table 3) were incubated in a humid chamber for 3 h at RT (except for ARF and HDM2, incubated overnight at 4°C). After washings with PBS, the respective secondary antibodies (Supplementary Table 3) were incubated for 1 h in a humid chamber at RT. Finally, the coverslips were mounted on glass slides containing VectaShield^®^ (Vector Laboratories). Samples were observed in a 63 × oil objective at LSM 510 (Zeiss) confocal microscope at the Analytic Central Facility (IQ-USP, São Paulo-SP, Brazil) and at LSM 780 (Zeiss) confocal microscope at INFAR (UNIFESP, São Paulo-SP, Brazil). All the visualization and acquisition analyses were performed with the Zen Blue or Black Lite software (Zeiss). The quantification of NPM and ARF proteins were made using the ImageJ software according to its tutorial. Briefly, each cell nucleus was manually contoured (according to the DAPI staining), and the total fluorescence of NPM staining present in the nucleus was quantified and assumed as 100%. All nucleoli were also individually contoured according to the NPM labeling (using anti-NPM antibody) on each of them and quantified by using the Image J. The fluorescence intensity of each subcellular compartment was used by the software to calculate the percentage of NPM present in nucleoli and/or nucleoplasm. The percentage of HDM2 present in the nucleoli was evaluated through colocalization with NAT10, a specific nucleolar protein used as marker that does not translocate out of the nucleolus after DNA damage (Zhang et al., 2015).

### Global RNA Transcription Rate by Ethynyl Uridine (EU) Assay

Nascent RNA staining was performed using the Click-iT^TM^ RNA Alexa Fluor^TM^ 488 Imaging Kit (Life Technologies), following the manufacturer’s protocol. Briefly, cells growing on round glass coverslips at 70% to 80% confluence were incubated with DMEM containing 2 mM 5-ethinyl-uridine (EU) (Invitrogen) at 37°C for 1 h. The cells were washed twice with PBS containing 2 mM EU and irradiated with UVC (6 J/m^2^ and 28 J/m^2^) or not (control group). Then, the cells were left to recover for 15 min with DMEM containing 2 mM EU. Coverslips were removed from the medium and fixed with a solution of 3% paraformaldehyde and 2% sucrose in PBS for 10 min at RT. After that, cells were washed twice with PBS (2 min at RT) and fixed again with methanol at −20°C for 20 min. After two more washes with PBS (5 min at RT), following the steps that were done according to the protocol Click-iT^®^ RNA Imaging Kit, coverslips were set up with Vectashield^®^ mounting medium supplemented with DAPI (VectorLabs). Images were captured in a Leica DMi8 fluorescence microscope, at 40 × magnification. For each condition performed in triplicates, the global RNA transcription was evaluated as a total fluorescence intensity of 100 individual nuclei measured by the ImageJ Software. The nucleoli were assumed as the area containing the higher fluorescence intensity that represents the hot spots of high RNA transcription rate.

### p53 Transcriptional Activity by Dual-Luciferase Reporter Assay

The two cell lines were seeded in white 96-well plate (1.5 × 10^4^ cells for MRC-5 or 3.5 × 10^4^ cells for XPA) in triplicate for each condition. One day after plating, a total of 105 ng plasmid DNA [100 ng pGL3-p53RE (p53 responsive element, or RE) containing the Firefly luciferase reporter, and 5 ng of pShuttle, containing the Renilla luciferase reporter] (Yu et al., 2007) were transfected using the transfection agent Lipofectamine 3000 (Life Technologies) according to the manufacturer protocol. After 24 h of transfection, cells were then irradiated (18 J/m^2^ UVC) and maintained in culture for a further 18 h when the Dual-Glo kit Luciferase Assay System (Promega) was used to measure the activity of the reporter genes. The reporter gene luciferase expression/activity was read using the luminometer Glomax-Multi Detection System (Promega). The data were then processed, where the Firefly luciferase signal was normalized for the Renilla luciferase signal, and this ratio was assumed as the percentage of levels of p53 transcriptional activity.

### Semi-Native Gel Electrophoresis

Semi-native gel electrophoresis was adapted from previously described for verification of NPM oligomerization (Hamilton et al., 2014). Cells were exposed to UV radiation (or not, control non-irradiated) and after 3 h were washed thrice with cold PBS and lysed on ice for 10 min in cold lysis buffer (50 mM Tris pH 8.0, 150 mM NaCl, 1% Triton X100, 1 mM EDTA, 1 mM EGTA, 50 mM NaF, 1 mM Na_3_V0_4_, 10 mM sodium β-glycerophosphate, 5 mM sodium pyrophosphate, proteases, and phosphatases inhibitors used as described). The lysates were rotated for 25 min at 4°C and centrifuged (21,000 × *g*, 15 min, 4°C). Quantification was done immediately, and 400 μg of a total protein was diluted in a sample buffer (62.5 mM Tris–HCl, 10% glycerol, 0.01% bromophenol blue) without boiling. Samples were loaded onto 10% Bis-Tris medium gel (1/3 Bis-Tris 1 M, acrylamide to 10% for resolving and 5% for stacking gel, plus APS and Temed). Gels were run (250 mM MOPS, 250 mM Tris, 5 mM EDTA, 0.5% SDS) at a constant voltage (50 V) at 4°C overnight and transferred to a nitrocellulose membrane and incubated as previously detailed for immunoblottings.

### Statistical Analysis

Graphs and statistical analyses were done in GraphPad Prism 8. All results are usually expressed as arithmetic average ± standard deviation (SD). Statistical comparisons between all groups were performed using ANOVA followed by a Tukey test with multiple comparisons. Values of *p* < 0.0001 (^****^), *p* < 0.001 (^∗∗∗^), *p* < 0.01 (^∗∗^) or *p* < 0.05 (^∗^) indicate statistical significance.

## Results

Dual-specificity phosphatase 3 was shown to have great influence on DNA repair capacity of cells exposed to genotoxic stress by UV (NER pathway) or IR (HR and NHEJ) (Torres et al., 2017; Russo et al., 2020). In these conditions DUSP3 physically interacts and colocalizes with nuclear proteins involved with DNA repair mechanisms, such as NPM (Panico and Forti, 2013). Therefore, this study aimed to show how DUSP3 interacts with and dephosphorylates NPM impacting on the p53 functions that include DNA repair, cell cycle, survival, and genomic stability in general.

**FIGURE 1:**
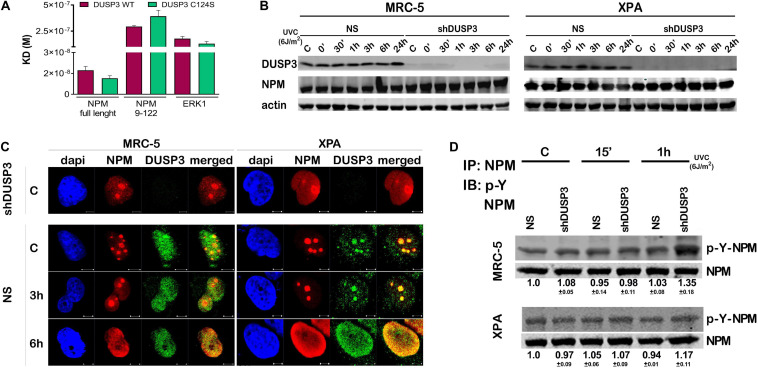
Dual specificity phosphatase 3 (DUSP3) binds with high affinity to nucleophosmin (NPM) *in vitro* and affects its nuclear localization and tyrosine phosphorylation. **(A)** The bimolecular interaction of purified recombinant proteins was performed by Surface Plasmon Resonance (SPR) and the obtained KD^#^ is shown in the graph. DUSP3 interacts with higher affinity to NPM full length compared to truncated NPM_9–12__2_, while ERK1 was used as classic control of DUSP3 substrate. **(B)** MRC-5 and XPA cell lines exposed to UVC radiation show NPM expression was not affected at all indicated times, regardless of the DUSP3 presence. **(C)** DUSP3 colocalizes with NPM before and after exposure to 18 J/m^2^ UVC. This colocalization occurs even after nucleoplasmic translocation of the NPM (complete kinetics is shown in Supplementary Figure 1). There is no signal of DUSP3 staining in the shDUSP3 cells by immunofluorescence. Representative images are only qualitative and white scale bars are 5 μM length at 63 × magnification. **(D)** Immunoprecipitation assays using NPM antibody and immunoblotted with anti phospho-Tyr antibody show an increase in the levels of phospho-Tyr-NPM in both MRC-5 and XPA shDUSP3 cells 1 h after UVC exposure. Immunoblottings are representative of experiments performed in triplicates, and the quantification is shown below each band as average ± standard deviation. ^#^ Note KD = ka/kd (M), where KD = equilibrium dissociation constant, ka = association rate constant, and kd = dissociation rate constant.

We started calculating the dissociation equilibrium constant (KD) between DUSP3 and NPM *in vitro* through the SPR technique: DUSP3-WT presented about 17 times higher affinity to full-length NPM than the truncated NPM_9–122_ (N-terminal domain) and 10 times higher affinity than its classical substrate ERK1, indicating that the entire tertiary and possibly the quaternary structures of NPM are necessary for the specificity and strength of this interaction. When we used the catalytically inactive DUSP3-C124S mutant, the KD values for the DUSP3-NPM interaction were still higher for full-length NPM than for the NPM_9–122_ (Figure 1A, Supplementary Figure 1A, and Supplementary Table 1). The calculated KD values for the DUSP3-NPM interaction *in vitro* are comparable and even higher than other bimolecular interactions from literature through SPR (Supplementary Table 2). When NPM expression was evaluated in MRC-5 (NER-proficient) and XPA (NER-deficient) cell lines, silenced for DUSP3 (using shDUSP3 that reduces DUSP3 in ∼95%) or not (using no silencing shRNA, NS) (Russo et al., 2020), with or without UV exposure, no differences were observed (Figure 1B). Confocal microscopy analyses showed that NPM and DUSP3 strongly colocalize within the cell nuclei before or after UV exposure. This colocalization was observed when NPM is inside the nucleolus before UV stress, or even when it completely translocated to nucleoplasm hours later (Figure 1C and Supplementary Figure 1B). Assays where NPM was immunoprecipitated with anti-NPM antibody and subsequently immunoblotted with specific anti-phospho-Tyr antibody showed a slight increase in tyrosine phosphorylation of NPM after UV exposure, which was still augmented under DUSP3 knockdown (Figure 1D). However, these experiments are not sufficiently accurate to determine which NPM tyrosine residue is specifically targeted by DUSP3 dephosphorylation, and other strategies were further employed.

**FIGURE 2:**
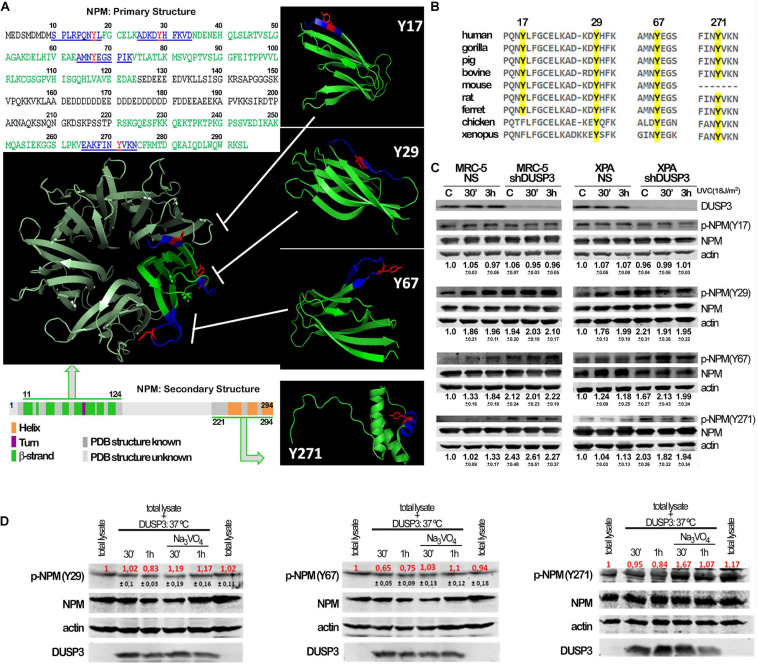
Conserved tyrosine residues of NPM are dephosphorylated by DUSP3. **(A)** The complete amino acid sequence of NPM protein shows the four tyrosine residues (in red: Y17, Y29, Y67, and Y271) inserted in decapeptide sequences (in blue) within the primary structure of NPM. The four tyrosine are localized in two regions of the primary and secondary structures of NPM (in green: one at very end N-terminal and the other at very end C-terminal), interspaced by a structurally unknown region (in gray), which have been crystallized and better studied. The four Tyr-containing decapeptide sequences were used as template to synthesize four decaphosphopeptides phosphorylated on each specific Tyr residue, which were used to immunization of rabbits and generation of phospho-specific antibodies. The oligomeric NPM structure (pentameric) proposed from functional studies (in metallic green; PDB 5EHD) was used as model to highlight the spatial position of the four tyrosines on NPM 3D structure (in green; PDB, 5EHD, and 2VXD). **(B)** Interspecies multiple alignment of the regions containing the four NPM tyrosines show these residues conservation throughout evolution. **(C)** Cellular lysates from MRC-5 and XPA cell lines (NS or shDUSP3) exposed to 18 J/m^2^ UVC radiation were immunoblotted using the antibodies against the four phospho-tyrosine residues of NPM. DUSP3 knockdown increased the phosphorylation of the 29, 67, and 271 tyrosines. **(D)** The *in vitro* dephosphorylation assays confirmed that DUSP3 can specifically dephosphorylate three tyrosine residues (29, 67, and 271), since their phosphorylation levels are decreased by the addition of exogenous DUSP3 to the lysates but are restored in the presence of Na_3_VO_4_. The immunoblotting images are representative of three independent experiments and the quantification is shown around the bands as mean (red) ± standard deviation (black).

Although not fully crystallized and understood, the NPM protein can homo-oligomerize in pentameric structures where we highlighted (in red) the four tyrosine residues along its sequence to indicate its potentialities as DUSP3 targets (Figure 2A). The four NPM tyrosines positioned as residues 17, 29, 67, and 271 are highly conserved along evolution (Figure 2B), from humans to *Xenopus*, which indicate their putative and still uncovered biochemical functions for the NPM protein. In this sense, we designed and synthesized four phospho-Y-decapeptides encompassing each one of the NPM tyrosines (in dark blue) (Figure 2A). Although Y17 is located within a β-sheet near the N-terminal, all four tyrosines are susceptible for binding and/or dephosphorylation by DUSP3, especially in the NPM monomers since this small phosphatase (only 21 kDa) harbors a very shallow active site. Each individual phospho-Y-decapeptide presented high binding affinity for DUSP3-WT in SPR experiments (Supplementary Figures 2A,B), and despite their lower KDs compared with the full-length NPM (Supplementary Table 1), these values indicate strong physical interaction and specificity to DUSP3, as can be seen for other classic peptide–protein bimolecular interactions (Supplementary Table 2). Next, the phospho-Y-decapeptides were used as epitopes to inoculate rabbits and to obtain four different polyclonal antibodies that specifically recognize each phosphorylated tyrosine on NPM structure (Supplementary Figure 2C). MRC-5 and XPA cell lines were exposed to UVC radiation and lysated 30 min and 3 h after for immunoblotting the phosphorylation profile of NPM tyrosine. Both cells presented detectable levels of p-NPM(Y17), p-NPM(Y29), p-NPM(Y67), and p-NPM(Y271) even at basal conditions (Figure 2C). However, after UV exposure, a systematic increase in the phosphorylation of Y29, Y67, and Y271 residues was observed and particularly augmented in shDUSP3 cells (Figure 2C). Similar results were also obtained for both cells exposed to 15 Gy of gamma radiation (Supplementary Figure 2D), suggesting that DUSP3 dephosphorylates these three tyrosines of NPM after other types of genotoxic stress. As a proof-of-concept, *in vitro* dephosphorylation experiments were performed in total lysates obtained from MRC-5 shDUSP3 cells collected 3 h after UV exposure, when NPM is maximally phosphorylated at Y29, Y67, and Y271. The addition of exogenous recombinant DUSP3-WT for 30 min and 1 h promoted dephosphorylation of Y29, Y67, and Y271 residues of NPM, whereas these reactions are reversed in the presence of Na_3_VO_4_ treatment, a potent pan inhibitor of PTPs (Figure 2D).

**FIGURE 3:**
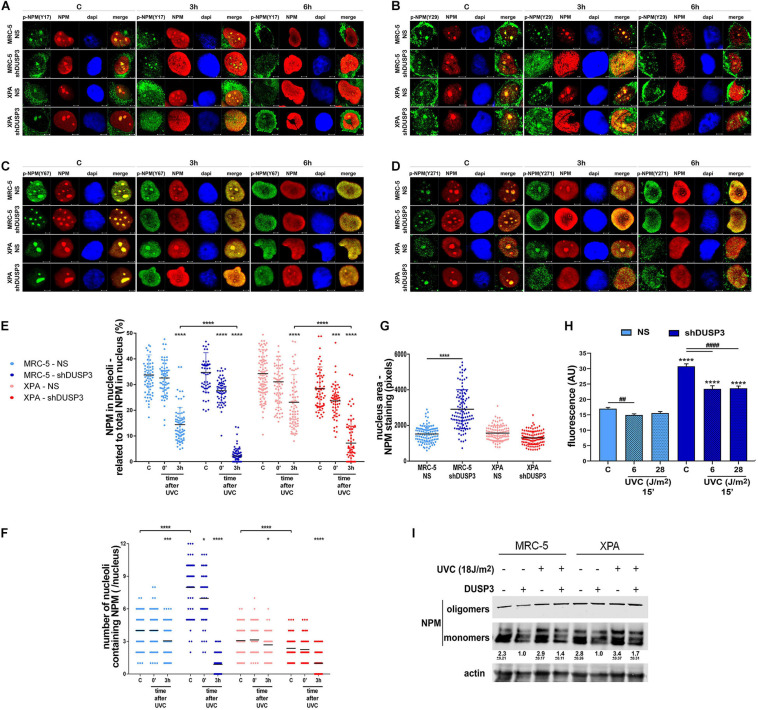
The NPM translocation and oligomerization, the global RNA transcription, and the nuclear and nucleolar morphology are all affected by DUSP3 knockdown. **(A–D)** To verify the location of Tyr-phosphorylated NPM after 18 J/m^2^ UVC exposure, confocal microscopy was performed in MRC5 and XPA cell lines (NS or shDUSP3) and compared with the staining of total NPM. The phosphorylation of Y29, Y67, and Y271 residues of NPM is observed in the nucleolus at basal conditions colocalizing with total NPM and remain phosphorylated after its translocation to the nucleoplasm (the complete kinetics is in the Supplementary Figure 3). In shDUSP3 cells p- Y29-, p- Y67-, and p-Y271-NPM reached the nucleoplasm 3 h after UVC, while in non-silencing (NS) cells they remain in nucleolus. Representative images are only qualitative and white scale bars are 5 μM length at 63 × magnification. **(E)** The NPM translocation was measured by ImageJ software as percentage of NPM present in nucleolus of at least 100 individual nuclei. shDUSP3 cells show an early nucleolus-nucleoplasm translocation of NPM. The same collected confocal images were used to count the number of nucleoli per nucleus **(F)** and the nuclear area **(G)**. In MRC-5 cells, the DUSP3 knockdown implied in greater number of nucleoli and larger nuclei compared to XPA cells. **(H)** General assay for RNA transcription using ethynyl uridine (EU) shows that MRC-5 shDUSP3 cells present greater transcriptional activity, size, and number of nucleoli per nucleus (Supplementary Figure 4). **(I)** Immunoblotting for NPM performed in gradient semi-native gels of total lysates from MRC-5 and XPA cells submitted or not to UV radiation. Representative blottings from three independent assays show greater levels of monomeric NPM under DUSP3 knockdown and after UVC exposure. Note: “–” indicates DUSP3 knockdown (shDUSP3 cells) and “+” indicates DUSP3 presence (NS cells). Anova: ****: *p* < 0.0001.

**FIGURE 4:**
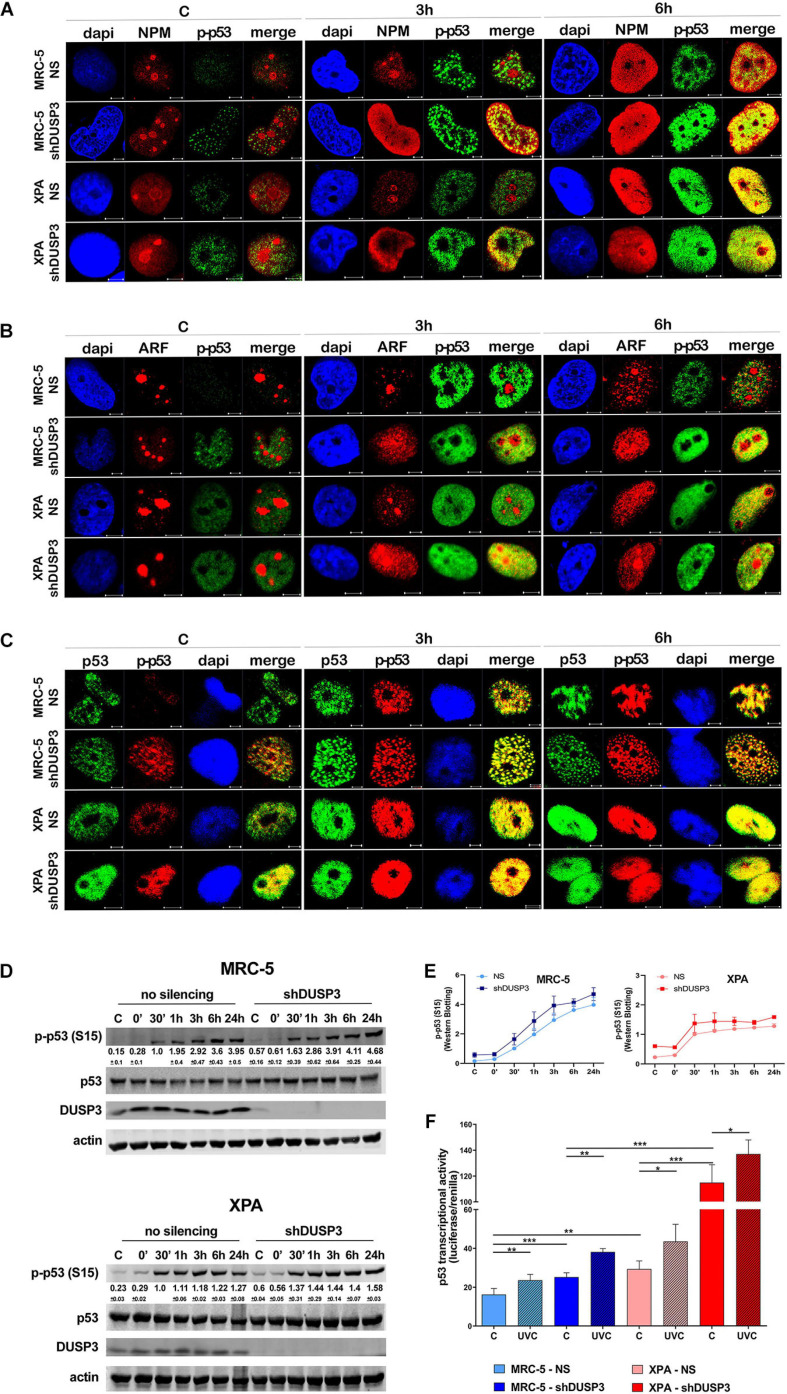
DUSP3 knockdown increases p53(Ser15) phosphorylation and p53 activity. MRC-5 and XPA cells were exposure to UVC radiation (18 J/m^2^) and submitted to confocal microscopy as indicated. **(A)** The p53 phosphorylation on Ser15 (p-p53) accompanies the nucleolus-nucleoplasm translocation of NPM: it peaks at 3 h after UVC exposure in shDUSP3 and it occurs at 6 h in NS cells. **(B)** The spatiotemporal colocalization of ARF and p-p53 occurs the same way as NPM and is also earlier in DUSP3 silenced cells. **(C)** Immunofluorescence images show p-p53 levels in DUSP3 knockdown cells before exposure to UV and peaking 3 h after stress, but only 6 h after in NS cells (complete kinetics are shown in Supplementary Figure 6). Representative images are only qualitative and white scale bars are 5 μM length at 63 × magnification. **(D)** The levels of p53(Ser15) phosphorylated are elevated in both DUSP3 knockdown cells compared to NS cells from 0 to 24 h after UV radiation. **(E)** The differences in p53(Ser15) phosphorylation caused by DUSP3 silencing were quantified and plotted from three independent experiments. **(F)** Firefly Luciferase gene reporter with a promoter containing p53 responsive element was transfected in cells and used to measure transcriptional activity of p53. Both MRC5 and XPA shDUSP3 cells exhibit greater p53 activity compared to NS cells, which is still higher in XPA cells. After exposure to UV radiation, p53 activity is increased in the control group and much more evidenced in DUSP3 silenced cells.

**FIGURE 5:**
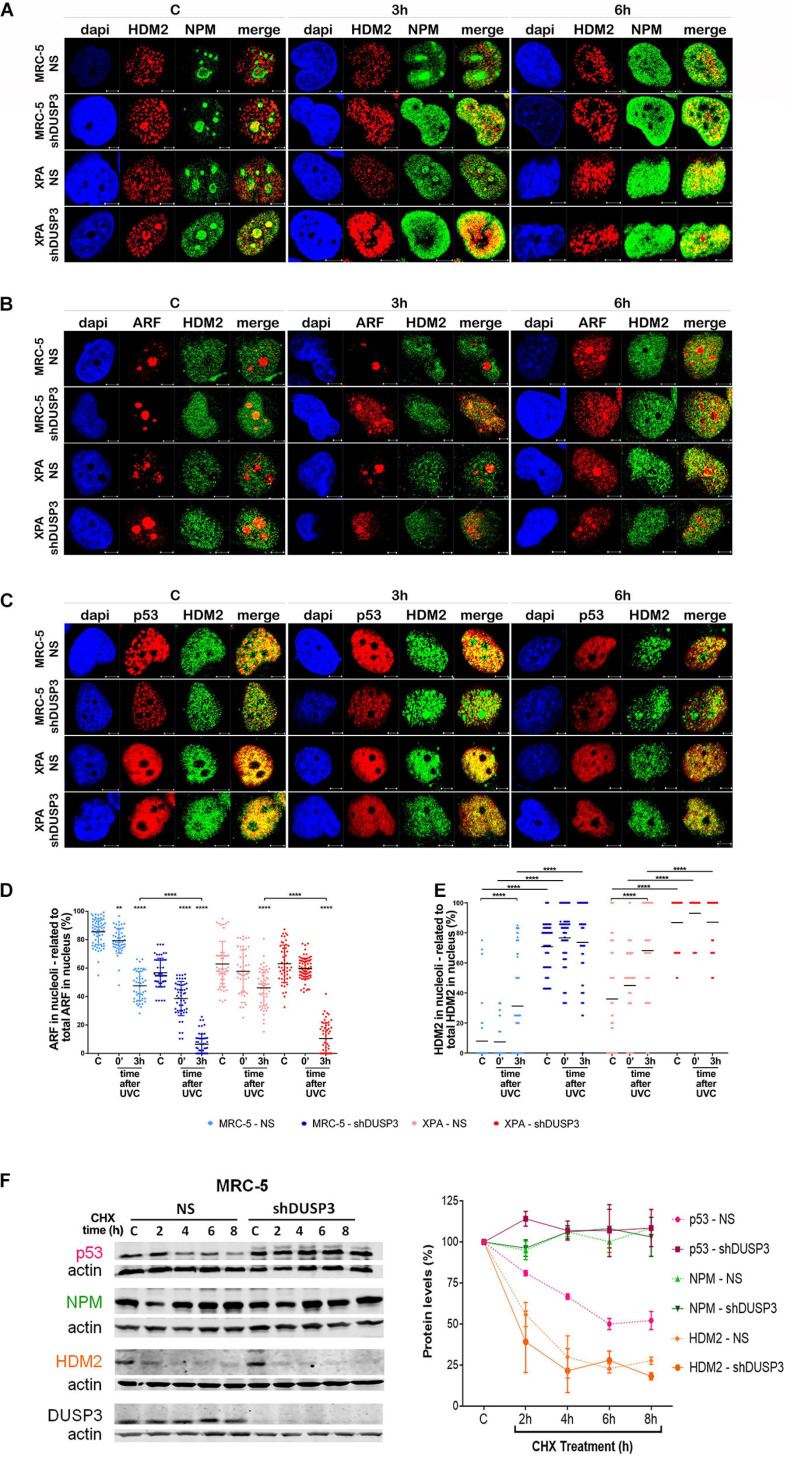
DUSP3 silencing relocates ARF and HDM2 in the nucleus and enhances p53 stability. MRC-5 and XPA cells were exposure to 18 J/m^2^UVC radiation and confocal microscopy was performed as indicated. **(A)** NPM colocalizes with HDM2 earlier (0 to 3 h) in shDUSP3 cells compared to NS (only in 6 h) after UV exposure. **(B)** Likewise, ARF colocalizes strongly with HDM2 as early as 3 h after exposure to UV in both DUSP3 silenced cells, while this colocalization is seen only in 6 h in NS cells. **(C)** The reduction of p53-HDM2 colocalization in the nucleoplasm is observed earlier in the shDUSP3 cells compared with NS controls. Representative images are only qualitative and white scale bars are 5 μM length at 63 × magnification. **(D)** Percentage of ARF protein present in nucleolus of at least 100 nuclei per condition measured using ImageJ and showing that ARF translocated earlier from nucleolus-to-nucleoplasm in DUSP3 knockdown cells. **(E)** The presence of HDM2 in nucleoli was measured by its colocalization with NAT10, a specific constitutive nucleolar marker, and it was expressed as percentage of nucleoli containing HDM2. Both MRC-5 and XPA shDUSP3 cells show high presence of HDM2 at nucleolus regardless UV radiation stress. Besides that, HDM2 is more retained in the nucleoli of XPA cells in basal conditions. **(F)** p53, NPM, and HDM2 proteins stability was verified in non-stressed MRC-5 cells by CHX treatments and followed by immunoblotting assays. Bands were quantified assuming the control condition (C = without CHX) of each cell line as 100% and normalized accordingly by the Actin loading control. An apparent increase in p53 protein stability is observed in MRC-5 shDUSP3 cells compared to NS cells. Blots are representative of three independent experiments. Anova: ***p < 0.001; ****p < 0.0001.

Once NPM translocates from the nucleolus to the nucleoplasm after DNA damage (Box et al., 2016) and that treatment with UV radiation and DUSP3 knockdown increase the levels of phospho-tyrosines (Figure 2C), we next investigated whether NPM translocation is influenced by its tyrosine phosphorylation (Figures 3A–D and Supplementary Figure 3). Confocal microscopy analyses showed that nucleolar NPM remains phosphorylated specially in p-NPM(Y29), p-NPM(Y67), and p-NPM(Y271) in basal conditions and during its translocation to nucleoplasm, which occurs after cell irradiation with UV (Figure 3B–D and Supplementary Figure 3B–D). The quantification of NPM translocation in cells expressing or not DUSP3 was measured in at least 100 individual nuclei for each condition, and it was expressed as the percentage of NPM present in the nucleolus related to the entire nucleus. The results showed an early translocation of NPM in 3 h caused by the DUSP3 knockout: in both shDUSP3 cell lines, NPM started translocating right after the UV treatment (0 min after UV) and is widely spread all over the nucleoplasm 3 h after (Figure 3E), while in NS cells, the nucleoplasmic NPM was detected only 6 h after UV radiation (Figures 1C, 3A–E, 4A, 5A). Concomitantly with NPM translocation, another observed and quantified phenotype was the greater number of nucleoli (Figure 3F) and the nuclear area (Figure 3G) of MRC-5 cells under DUSP3 knockdown compared with NS or even XPA cells. To corroborate these findings, measurements of global RNA transcription by 5-ethynyl uridine (EU) revealed that MRC-5 shDUSP3 cells have greater ribosomal RNA staining in the nucleolus compared with NS cells. That means DUSP3 knockdown increased the global RNA transcription rate, which dropped after UV treatment (Figure 3H and Supplementary Figure 4A). Moreover, the EU method showed an elevated number of nucleoli (Supplementary Figure 4C) and the nuclear area (Supplementary Figure 4B) in MRC-5 shDUSP3 cells. To possibly intersect these results of changes in nuclear morphology and transcription rate with those of NPM phosphorylation and translocation, we looked for differences in monomer and oligomeric NPM levels in both cells, with and without UV stress, through semi-native gradient gels (Figure 3I). Higher levels of NPM monomers were measured in MRC-5 shDUSP3 cells compared with those in NS cells, especially 3 h after UV exposure. These results suggest that tyrosine phosphorylation of NPM impacts on the equilibrium of monomers ↔ oligomers displacing it for the disassembly of pentameric structures and favoring the translocation of NPM monomers out of the nucleolus after stress (Figure 3I).

It is known that NPM interacts with ARF in the nucleolus, and after DNA damage, both can translocate to the nucleoplasm and to cause disruption of p53-HDM2 interaction (Kurki et al., 2004b), which allow p53 stabilization and activation by, for example, phosphorylation in Ser15 (Loughery et al., 2014). For this purpose, confocal microscopy was performed to verify spatiotemporal colocalization between these proteins (Figure 4 and Supplementary Figure 5). The results demonstrated that NPM colocalizes with p-p53 as early as 3 h after UV exposure in both MRC-5 and XPA shDUSP3 cells, but only at 6 h in NS cells (Figure 4A and Supplementary Figure 5A). Similarly, and at the same time-points, ARF also colocalized earlier with p-p53 at the nucleoplasm in both DUSP3 knockdown cells, while the nucleus of all cells resembles at 6 h after UV radiation (Figure 4B and Supplementary Figure 5B). Confocal assays also demonstrated high levels of p53 (Ser15) phosphorylation in basal conditions (not irradiated) of both shDUSP3 cells compared with those in NS cells, which still increase after UV exposure and especially under DUSP3 knockdown. However, both NS and shDUSP3 cells present comparable levels of total p53 at nucleoplasm regardless UV radiation (Figure 4C and Supplementary Figure 5C), although XPA cells are known to express higher levels of p53 (Carvalho et al., 2008). These immunocytochemistry results were quantitatively confirmed by immunoblotting assays (Figures 4D,E) that showed higher levels of p-p53 in shDUSP3 cells before and principally after UV treatments. To test whether the increase in p53(Ser15) phosphorylation reflects in high transcriptional activity, a Firefly Luciferase reporter gene, controlled by a responsive element (RE) bound and activated by p53, was transfected into these cell lines. Corroborating the previous data, the basal activity of p53 was approximately twofold in XPA cell lines compared with that in MRC-5, and in both cell lines under DUSP3 knockdown, the p53 activity almost doubled (in MRC-5) or triplicated (in XPA) compared with that in NS cells. In addition, as already expected for DNA damaging that promotes p53(Ser15) phosphorylation, after UV radiation, the levels of active p53 were elevated and even higher in DUSP3-silenced cells (Figure 5F).

For a better understanding, the molecule regions involved in the interactions between NPM, ARF, MDM2, and p53 were schematically presented and highlighted among many other major domains (Supplementary Figure 6). According to that, extensive series of confocal microscopy were performed to evaluate NPM and ARF colocalization with HDM2 in the nucleoplasm of MRC-5 and XPA cells (Figure 5A and Supplementary Figures 5B,7A,B, respectively). The results of nucleoplasmic colocalization of HDM2-NPM were in good agreement with previous results of NPM translocation (Figures 3A–E, 4A, 5A, and Supplementary Figure 7A). The nucleolus–nucleoplasm translocation of ARF occurs as early as 3 h after UV radiation in both MRC-5 and XPA shDUSP3 cells, although only 6 h later in NS cells (Figures 4B, 5B and Supplementary Figure 7B). In addition, the stronger colocalization between ARF-HDM2 can also be observed 3 h after UV radiation in MRC-5 and XPA shDUSP3 cells, whereas after 6 h, all cells behave the same (Figure 5B and Supplementary Figure 7B). The advanced nucleoplasmic translocation of NPM and ARF anticipated the diminution of colocalization between p53-HDM2 in MRC-5 and XPA shDUSP3 cells even before UV treatment, while it remained intense until almost 6 h after UV in NS cells (Figure 5C and Supplementary Figure 7C). ARF translocation was quantified by estimating the percentage of protein present in the nucleolus *versus* nucleoplasm, and just like for NPM, there was a 3-h lag in the ARF translocation in NS cells compared with that in shDUSP3 cells (Figure 5D). Another interesting observation from confocal analyses was an unexpected presence of HDM2 in the nucleoli of cells (Figures 5A–C and Supplementary Figures 7A–C). Therefore, by using the NAT10 protein, a constitutive nucleolar marker (Zhang et al., 2015), we quantified the levels of HDM2 in the nucleoli of both cells with and without DUSP3 knockdown, before or after UV radiation (Figure 5E). In NS cells, the nucleolar level of HDM2 was only increased 3 h after UV treatment, meaning that under DUSP3 presence, HDM2 is more nucleoplasmic than nucleolar. However, in the absence of DUSP3 for both cells, HDM2 presented a preferential nucleolar retention, apparently independent on the UV stress. These results also coincided with the reduced colocalization between HDM2-p53 in the nucleoplasm of shDUSP3 cells (Figure 5C). In the same sense, we seek to investigate the stability of p53, NPM, and HDM2 proteins under the presence of cycloheximide (CHX). The most important result was an increase in p53 stability up to 8 h after CHX treatment in DUSP knockdown cells, while in NS cells, the p53 degradation occurred in less than 2 h. There were only slight differences in the NPM and HDM2 stability between shDUSP3 and NS cells, and although NPM appeared as a very stable protein, HDM2 presented a remarkably high turnover, as expected (Figure 5F).

## Discussion

The loss of DUSP3 negatively impacts on genomic stability mechanisms through the modulation of DNA repair pathways such as DDR, HR, NHEJ, and more recently, NER (Forti, 2015; Torres et al., 2017; Russo et al., 2020). DUSP3 also affects the expression of certain cyclins, CDKs, and p21^Cip1^ proteins to regulate cell cycle and proliferation of cells exposed to UV radiation (Russo et al., 2020). However, none of the classic DUSP3 substrates (Alonso et al., 2001; Hoyt et al., 2007; Wang et al., 2011; Chen et al., 2017) could explain these new biological functions, especially considering that uncommon interactors are indirectly involved with different chromatin functions, including remodeling, replication, and repair (Panico and Forti, 2013). Among these protein partners, NPM was the focus of this work since, besides its large involvement in DNA repair pathways and under different stimuli, the post-translational regulation of NPM remains unexplored under the prism of genomic stability. Despite being considered a phosphoprotein with phosphorylations on threonine 199, 234, and 237 that allow functions in centriole duplication (Chan and Lim, 2015), and in the expression of DNA repair genes (Koike et al., 2010; Kyheröinen and Vartiainen, 2019; López et al., 2020), the identification of tyrosine phosphorylation on NPM and its biochemical functions have not yet been explored.

Nucleophosmin continuously shuttles between nucleolus, nucleus, and also cytoplasm (Borer et al., 1989) to regulate ribosome biogenesis, mRNA processing, centrosome duplication, and genome stability through chromatin remodeling and DNA repair (Box et al., 2016). NPM is a very stable protein in many cellular contexts and can have increased expression in cells exposed to UV (Wu and Yung, 2002), or decreased in immune cells under DUSP3 knockdown when NPM is more degraded due to high levels of STAT5(Y694) phosphorylation (Ren et al., 2016). The interaction between DUSP3-NPM was already demonstrated *in cells* exposed to genotoxic stress (Panico and Forti, 2013), and here we show *in vitro* that DUSP3 has very high binding affinity to NPM, even stronger than the DUSP3-ERK1 interaction, a classic substrate of this phosphatase in *in vitro* and *in vivo* studies (Alonso et al., 2001). NPM has four tyrosine residues (17, 29, 67, and 271), all presenting high probability of phosphorylation according to different bioinformatics prediction tools and to proteomic and phosphoproteomic databases (Supplementary Figure 8). These NPM tyrosines are in regions previously characterized by X-ray crystallography^2^ and are conserved among many species (Mitrea et al., 2014). According to these findings, we demonstrated that NPM is tyrosine-phosphorylated especially in cellular conditions of genotoxic stress. Considering its shallow catalytic site, strong colocalization with, and high affinity for, NPM, we demonstrated that DUSP3 is a phosphatase candidate to dephosphorylate NPM tyrosines. This is the case of those sterically available residues, particularly the last three ones (29, 67, and 271) located in more exposed sites without the hindrances imposed by the oligomeric structure. Therefore, we hypothesized NPM as the missing link between DUSP3 and DNA repair pathways, and hence, we investigated how its tyrosine phosphorylation would impact on cellular responses after DNA damage.

It is known that NPM translocates from the nucleoli to the nucleoplasm normally starting not less than 3 h after UV radiation (Kurki et al., 2004b), in a process that may be dependent on the proteasome activity (Moore et al., 2013). Our results in both DUSP3-proficient cells MRC-5 and XPA agree with that. However, we found that under DUSP3 knockdown, NPM starts translocating earlier, right after the UV irradiation (radiation exposure followed by immediate cell collection = time 0 min = 0’) and accumulates in the nucleoplasm even before 3 h after UV stress. ARF presents the same translocation kinetics as NPM in shDUSP3 cells, since the interaction between ARF and NPM is disrupted after DNA damage and triggers their individual translocation out of nucleoli (Lee C. et al., 2005; Moore et al., 2013), whereas this interaction is required for nucleolar localization of ARF (Lee et al., 2017). The absence of DUSP3 causes massive and premature NPM translocation to the nucleoplasm here suggested to be caused by the increased phosphorylation of tyrosines 29, 67, and 271 (that remains phosphorylated after NPM translocation), which indirectly may be favoring a similar ARF translocation. The latter happens because the C-terminal region of ARF, which corresponds to its predicted nucleolar localization signal, interacts with the N-terminal domain of NPM (residues 16–123) (Luchinat et al., 2018) that encompasses two of these tyrosines (Y29 and Y67). NPM and ARF proteins can bind to HDM2 and p53 in the nucleoplasm (Kurki et al., 2004a,b; Lee C. et al., 2005), whereas phospho-tyrosine-NPM might present a different affinity for interacting with HDM2 at the nucleoplasm or even at the nucleolus under DUSP3 absence. The nucleoplasmic interaction ARF-HDM2 unleashes the nucleolar localization signal present in the C-terminal of HDM2 facilitating its sequestration to the nucleolus, therefore contributing to prevent or mitigate the p53 ubiquitination (Lohrum et al., 2000). The C-terminal half of NPM is important for its interaction with both HDM2 and p53, and also for nucleolar localization (Lambert and Buckle, 2006; Grummitt et al., 2008; Luchinat et al., 2018) since it contains the nucleolar localization signals assigned by the W288 and W290 residues (Falini et al., 2006). Accordingly, the Y271 of NPM is hypothetically another important site undergoing phosphorylation/dephosphorylation cycle that influences the nucleolus–nucleoplasm shuttling of NPM.

The greater p53(S15) phosphorylation in DUSP3 knockdown cells, even without stress, is possibly facilitated by an earlier translocation or sequestration/recruitment of HDM2 toward the nucleoli. After UV exposure and especially under DUSP3 absence, the levels of p53(Ser15) phosphorylation peak hours in advance coincidently with the early NPM and ARF translocation to the nucleoplasm. The phosphorylation of p53(S15) is particularly known to block the E3 ubiquitin–ligase activity of HDM2 (Dai and Gu, 2010), a positive feedback mechanism that releases HDM2 in the nucleoplasm and allows its binding to NPM and ARF, thus making possible its rapid nucleolar localization. NPM translocation is a process dependent on changes in its quaternary structure: whenever in the nucleolus, NPM is found assembled into two pentameric structures connected head-to-head (Lys80 of NPM makes a direct hydrogen bond to Asp55 of the opposing subunit) (Hyung et al., 2007). The homo-oligomeric NPM plays its main roles at nucleolus functioning as a histone H1 chaperone to promote chromatin remodeling by compaction and by diminishing global RNA transcription (Lindström, 2011; Elsässer and D’Arcy, 2012). On the other hand, the increase in NPM translocation out of nucleolus is associated with its availability in monomers (Kim et al., 2015), which is also associated with increased levels of p53 phosphorylation and induction of apoptosis (Holoubek et al., 2018). It was demonstrated that phosphorylation of threonines at the N-terminal of NPM favors the equilibrium toward NPM monomeric forms, but this effect can be reversed when NPM interacts with different partners in the nucleoli (Mitrea et al., 2014). All these molecular mechanisms are somehow recapitulated by the results raised in this work for both cell lines under DUSP3 knockdown. For example, our results suggest that under DUSP3 absence, the Y29 and Y67 residues present in the N-terminal region of NPM remain phosphorylated and, therefore, can also displace the equilibrium toward monomeric NPM to facilitate the NPM–ARF interaction and/or their subsequent nucleoplasmic translocation. Another interesting outcome is once XPA naturally have greater levels of p53 than the MRC-5 cells, HDM2 protein is already found in the nucleolus at basal conditions, which is further increased by UV stress and exceptionally elevated under DUSP3 silencing. These results can be associated with the increased levels of p21^Cip1^, G1/G2 arrest, and cell death (apoptosis and senescence) in NER-proficient or NER-deficient cells submitted to DUSP3 knockdown, as we recently demonstrated (Torres et al., 2017; Russo et al., 2020). These cellular phenotypes, marked by a reduced cell cycling, proliferation, and survival, may also be caused by the increased p53 stability and phosphorylation at Ser15 due to NPM phosphorylation in the Y29, Y67, and Y271 residues. Other studies also reported that negative regulation of NPM functions causes ARF translocation to the nucleoplasm and upregulation of p21^Cip1^ expression through the p53 stabilization and inhibition of cell proliferation (Wang et al., 2016). Finally, the DUSP3 knockdown increases transcriptional activity of p53 and the overall transcription rate of RNA, which correlate with the largest size and number of nucleoli, as well as the biggest cell nucleus size. Altogether, these results support the hypothesis that tyrosine phosphorylation of NPM impinges its chaperone activity upon histone H1, relaxing the chromatin to favor nucleolar rRNA transcription and global transcription (Murano et al., 2008; Gadad et al., 2011).

Nucleophosmin is also necessary for p53 tetramerization and stabilization since its tetramer structure is essential for its site-specific DNA binding, specific protein–protein interactions (PPI), and post-translational modifications (PTM) (Kamada et al., 2016). In this sense, the DUSP3 knockdown might enhance the p53 tetramer formation by increasing its half-live through allowing an advanced NPM interaction with p53-HDM2, what will culminate in its greater activity, and considering that phospho-tyrosine NPM translocates earlier and faster from nucleolus, we can speculate that this nucleoplasmic NPM is promptly redirected to functions other than ribosome biogenesis, such as transcription regulation of DNA repair genes by acting in histone H1 folding and chromatin remodeling (Lindström, 2011; Box et al., 2016). In this sense, knocking down DUSP3 in cells promotes a markedly worsening of DNA damage response and repair after UV or ionizing radiation exposure, as our group has demonstrated (Torres et al., 2017; Russo et al., 2020).

**FIGURE 6:**
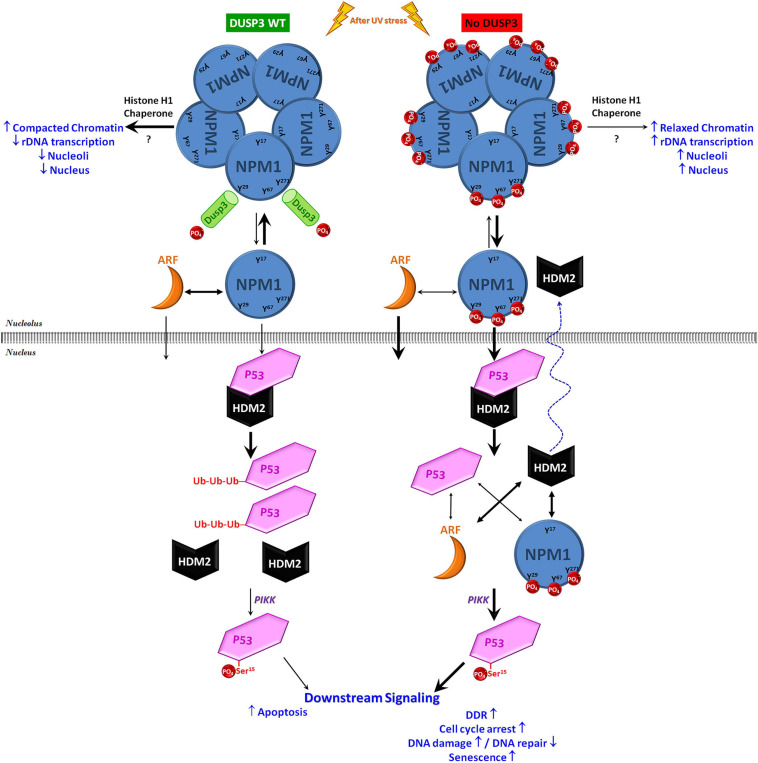
Schematic model on the contribution of DUSP3-NPM axis to p53 actions in the maintenance of genomic stability. Under conditions of cellular homeostasis, the NPM present in the nucleoli is constantly interacting with and being dephosphorylated by DUSP3 on residues Y29, Y67, and Y271. In the absence of DUSP3, these three residues remain phosphorylated and favor the dissociation equilibrium of NPM homo-oligomerization and/or its association with ARF, therefore promoting an early nucleoplasmic translocation of monomeric NPM and ARF. Once in the nucleoplasm, these two proteins can induce the dissociation of the HDM2-p53 interaction through the individual binding to one or the other protein. This mitigates the process of p53 degradation (via proteasome), increasing its half-life and, therefore, allowing its phosphorylation in Ser15 (through kinases of the PIKK family) that subsequently increase its transcriptional activity. Therefore, as previously reported in a DUSP3 deficiency scenario, the greater p53 activation modulates the downstream pathway to regulate cellular responses to genotoxic stress, causing cell cycle arrest associated with the absence or insufficient DNA repair, followed by senescence and reduced cell proliferation/survival (Torres et al., 2017; Monteiro et al., 2019; Russo et al., 2020).

In conclusion, this work hypothesizes that in normal cellular backgrounds, DUSP3 is constantly interacting and dephosphorylating NPM on its Y29, Y67, and Y271 residues culminating in controlled levels of p53 to drive cell cycle checkpoints, DNA repair, and cell death. In the absence of DUSP3, phospho-tyrosine NPM is preferably found as monomers that translocate early out of the nucleolus together with ARF proteins. Once at the nucleoplasm, these proteins compete to binding HDM2 or p53, reduce the HDM2-p53 interaction, and mitigate p53 degradation. This allows p53 phosphorylation at the Ser15, increasing its half-life and transcriptional activity upon p53 responsive elements (Figure 6). To corroborate these molecular mechanisms, we found other proteins up- or downregulated by DUSP3 [(Russo et al., 2020; Pereira et al., 2021); Luna ACL & Forti FL, unpublished results] that are encoded by p53-regulated genes (Fischer, 2017) that might explain the anti-proliferative phenotype of DUSP3 knockdown cells. Thus, the DUSP3-NPM-p53 signaling axis brings new insights for knowledge about the p53 pathway regulation, which remains of great interest in cancer therapeutics. Novel mechanisms by which the nucleolus controls cellular stress responses are under intense scrutiny, as recently published in the NPM deacetylation regulating p53 stability under UV-induced DNA damage (Ianni et al., 2021). This is another piece of the intriguing puzzle under the NPM-p53-HDM2 axis, as well as this manuscript; however, since DUSP3 seems to be the “eraser” of the NPM phospho-tyrosines, which “writer” kinase is responsible for their phosphorylation is an issue that needs further investigation.

## Data Availability Statement

The original contributions presented in the study are included in the article/Supplementary Material, further inquiries can be directed to the corresponding author/s.

## Author Contributions

LR and PF performed this study. FF supervised and designed the study. LR, PF, and FF analyzed and interpreted the data. LR and FF wrote the manuscript. All authors contributed to the article and approved the submitted version.

## Conflict of Interest

The authors declare that the research was conducted in the absence of any commercial or financial relationships that could be construed as a potential conflict of interest.

## Footnotes

1 https://www.rostlab.org/services/epitome/

2 www.rcsb.org
