# Supplementary material for: Nucleophosmin Protein Dephosphorylation by DUSP3 Is a Fine-Tuning Regulator of p53 Signaling to Maintain Genomic Stability
Source: Front Cell Dev Biol. 2021 Mar 11;9:624933. doi: 10.3389/fcell.2021.624933 (PMC7991746; doi:10.3389/fcell.2021.624933)
Supplement: Supplementary file 1 [file Data_Sheet_1.docx]

**SUPPLEMENTARY MATERIALS**

**FIGURES**

**Figure S1**


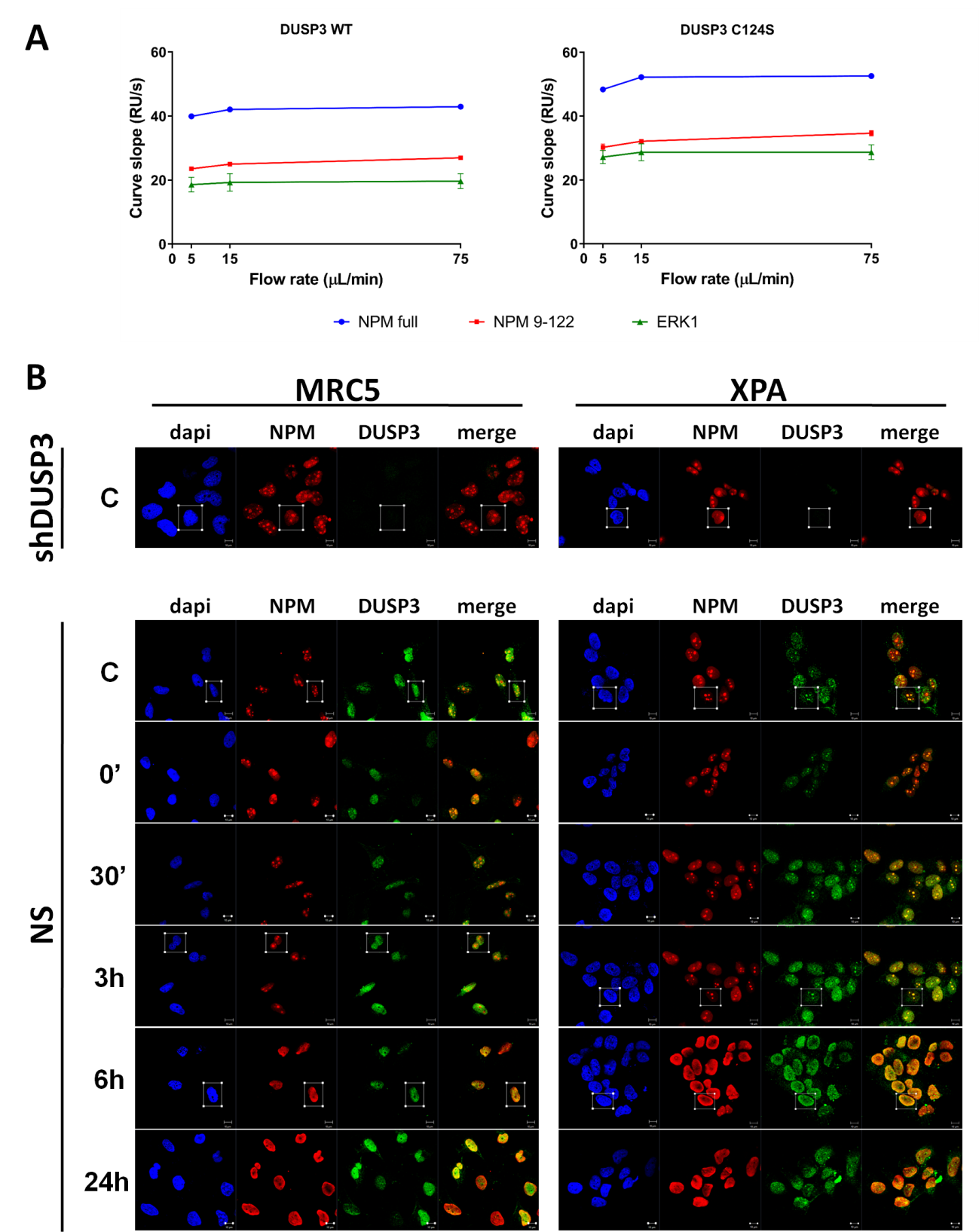


**Fig. S1. DUSP3 interacts and colocalizes with NPM at the nucleus.** **(A)** The bimolecular interaction between DUSP3 (WT and C124S) and NPM using the Biacore equipment were carefully followed by running control tests to calculate constant of the mass-transfer rate on the same sensorchips. Graphs confirmed that the established and standardized method employed was able to dissociate every protein after binding in a reliable manner. There was no excess of immobilized protein, and no difference in the interaction values according to the injection volumes, assuring that the results showed in the Fig. 1 were not caused by spurious mass transfer. **(B)** MRC-5 and XPA cells non-silenced for DUSP3 were submitted to 18 J/m2 UVC radiation and collected for immunofluorescence staining after 0 min, 30 min, 3 hours, 6 hours, and 24 hours. DUSP3 colocalizes with NPM in all time-points and so remains even after NPM translocation out of nucleolus to nucleoplasm. There was no signal of DUSP3 staining in the shDUSP3 cells. The white squares show the representative cell showed in the Fig. 1 and white bar scale indicate 10 µm length at 63x magnification.

**Figure S2**


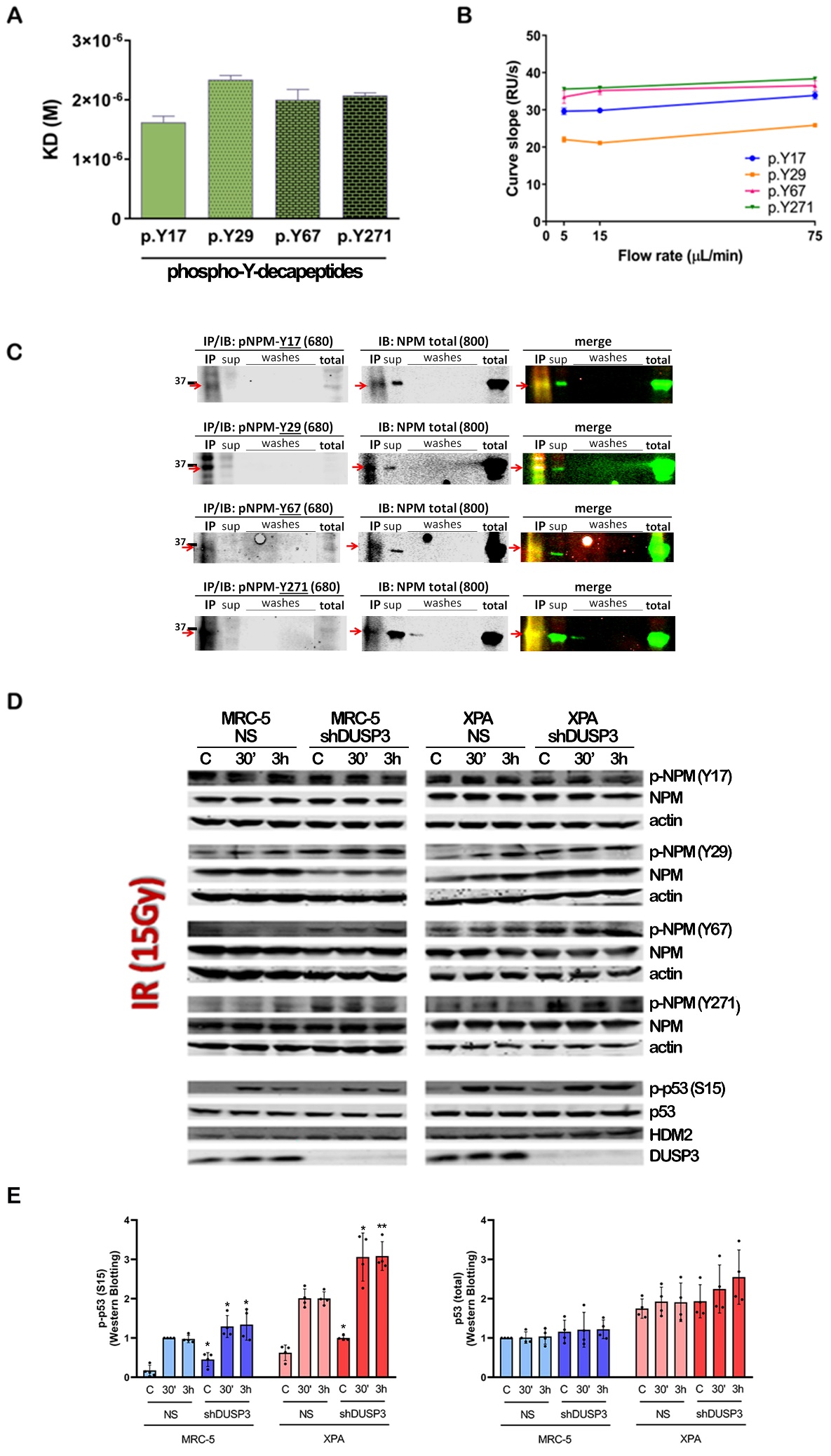


**Fig. S2. DUSP3 interacts with phospho-Y-decapeptides of NPM *in vitro* and dephosphorylates phospho-Y-NPM *in cells*.** (**A)** The four phospho-Y-decapeptides designed for production of specific antibodies against NPM tyrosines were assayed by SPR for evaluating the bimolecular interaction with DUSP3-WT. DUSP3 interacted with high affinity to the peptides and KD values were comparable to other common peptide-protein interactions of literature (Supplementary Table 2). **(B)** The obtained KD values from A were significant are real, and therefore not influenced by eventual spurious mass transfer. **(C)** Immunoprecipitation assay was used to verify the specificity of the antibodies against the phosphorylated NPM tyrosines. 200 µg of a total lysate of MRC-5 NS was individually incubated with each antibody for 18 hours at 4 °C under rotation (in the presence of proteases/phosphatases inhibitors). The complexes were incubated with A/G Plus-Agarose Protein for 1.5 hours under rotation at 4° C and washed 5 times with RIPA in the presence of proteases/phosphatases inhibitors. After SDS-PAGE, the membranes were firstly immunoblotted with the same antibodies against p-NPM (17, 29, 67 or 271) and then revealed with the secondary rabbit antibody IR Dye 680 nm. Subsequently, the membranes were incubated again but using antibody against total NPM and then revealed with the secondary mouse antibody IR Dye 800 nm. Merged images reveal the superposition of the immunoblottings, where the yellow color indicates the specificity of these antibodies for tyrosine phosphorylated residues of NPM protein (green is total NPM and red is phospho-Y-NPM, here shown in gray tons). **(D)** To corroborate the results of Fig. 2 we tested the DUSP3 dephosphorylation over the four NPM tyrosines (17, 29, 67 and 271) after treating the cells with 15 Gy gamma radiation for 30 min or 3 hours. DUSP3 knockdown raises the phosphorylation levels of p-NPM(Y29), p-NPM(Y67) and p-NPM(Y271) raised by the IR. And again, phosphorylation of p53(Ser15) was increased in shDUSP3 cells compared to NS cells, even in control or treated conditions. In agreement, a slight increase in the levels of HDM2 was observed. The images are representative of three independent experiments in triplicate. **(E)** Quantification of p-p53(Ser15) and total p53 levels from panel D. The p53(Ser15) phosphorylation in the different times and cells was compared to the phosphorylation of the MRC-5 NS cells after 30’ IR. Once more, both shDUSP3 cell lines presented higher p53(Ser15) phosphorylation in the control and after IR treatment. *: p<0,05, **: p<0,001 compared to the NS cells at respective times. The levels of the p53 total protein were normalized to control MRC-5 NS cells and there were no significative differences between NS and shDUSP3 cells.

**Figure S3**


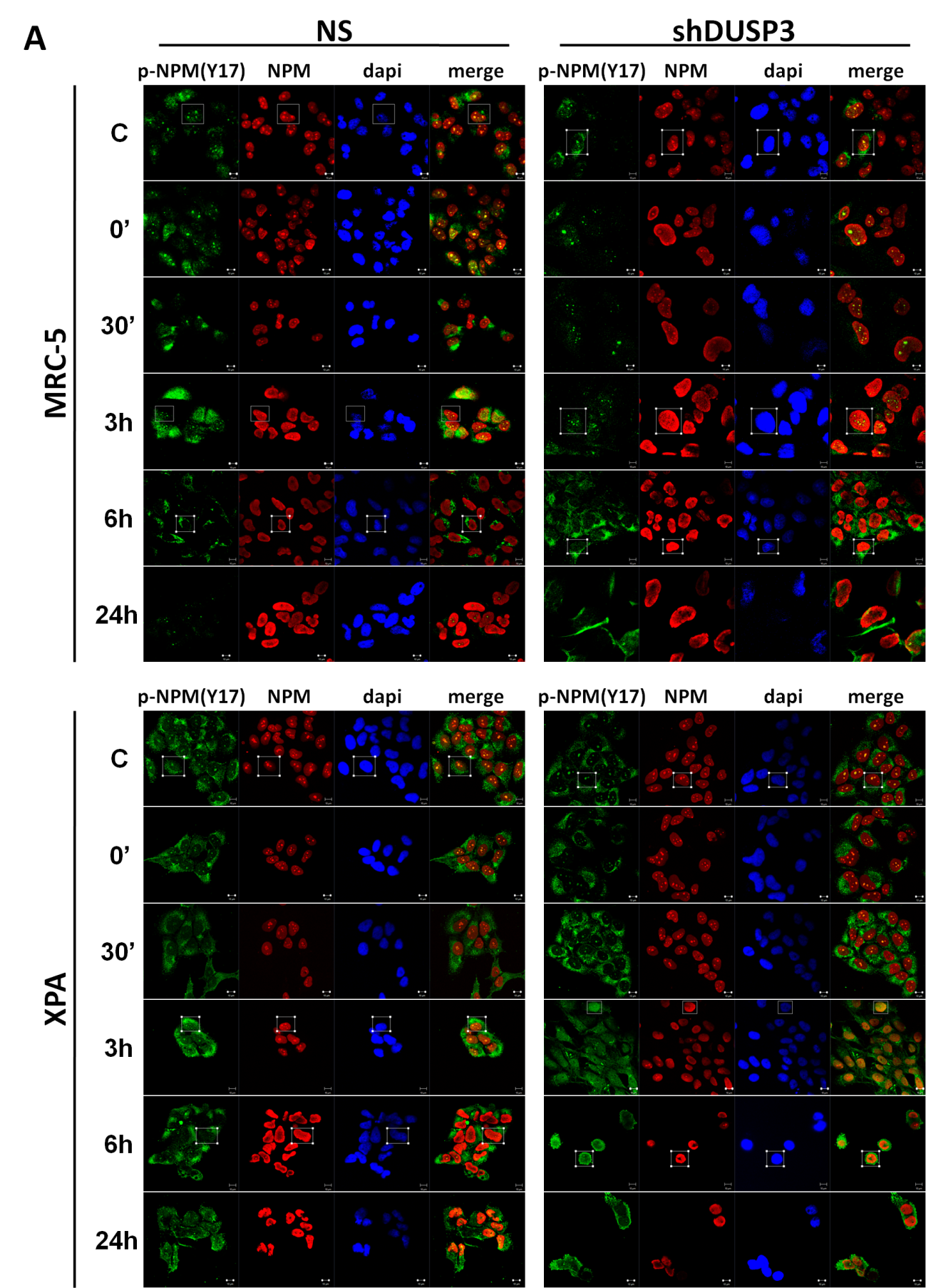


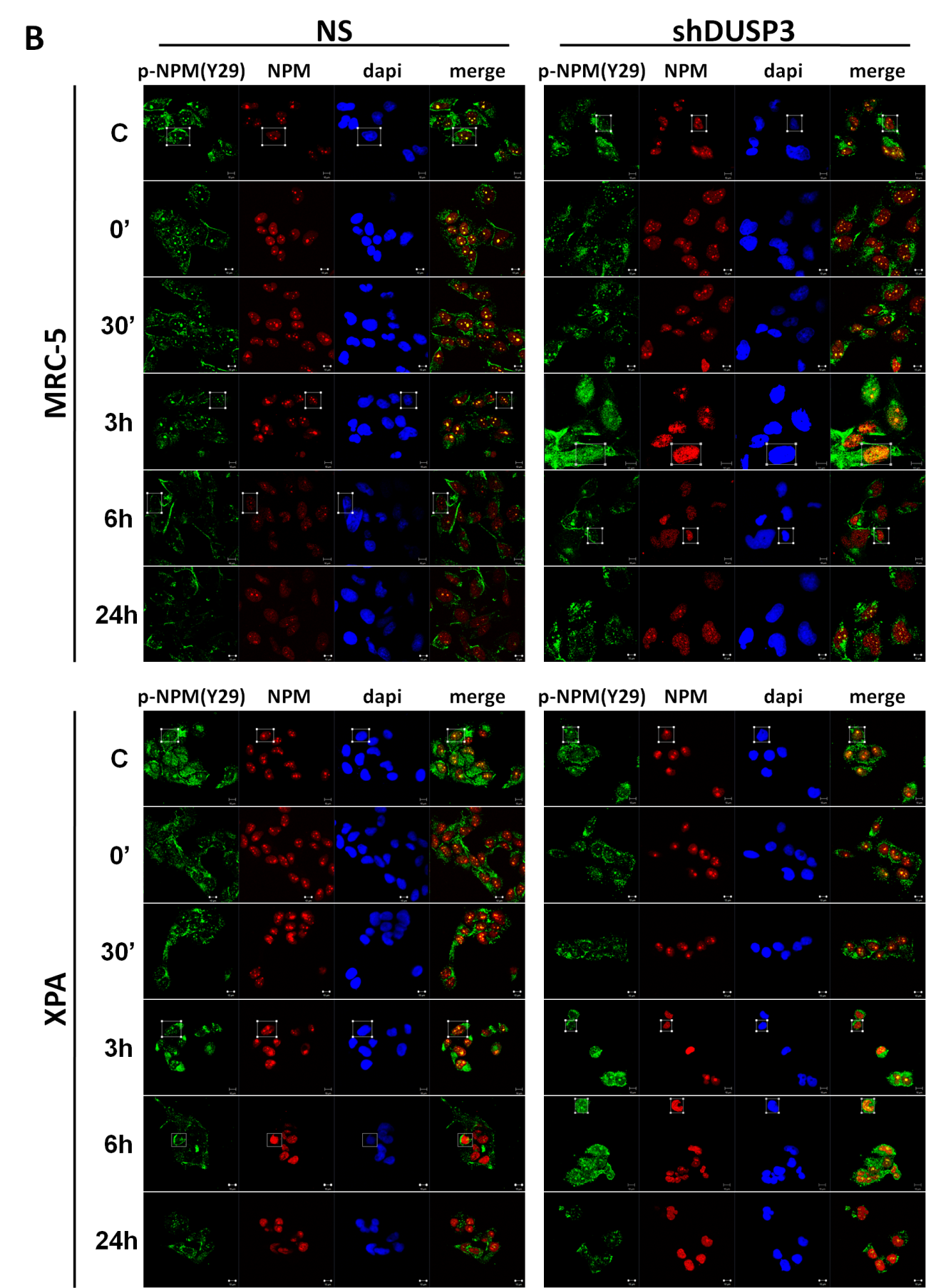


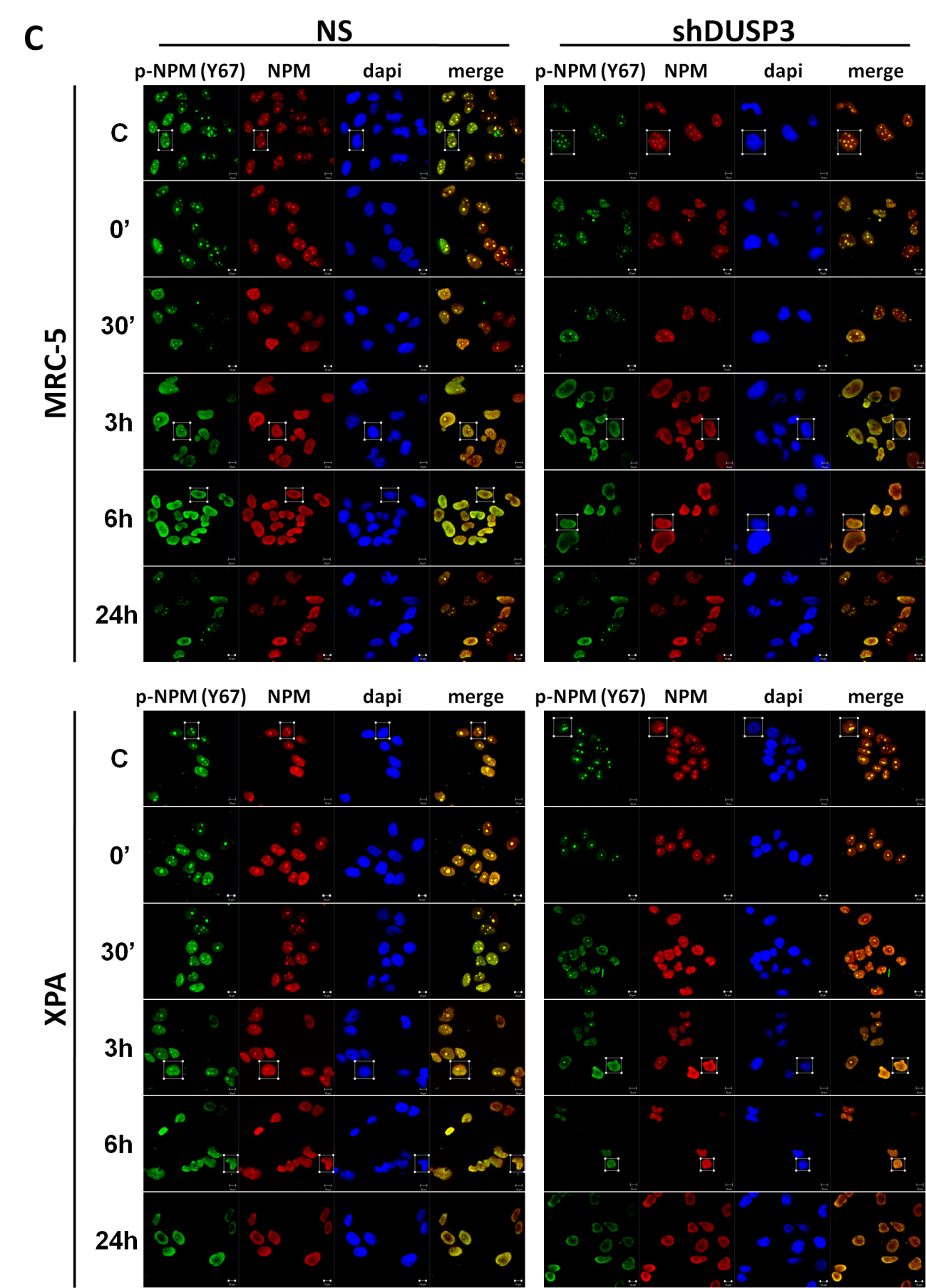


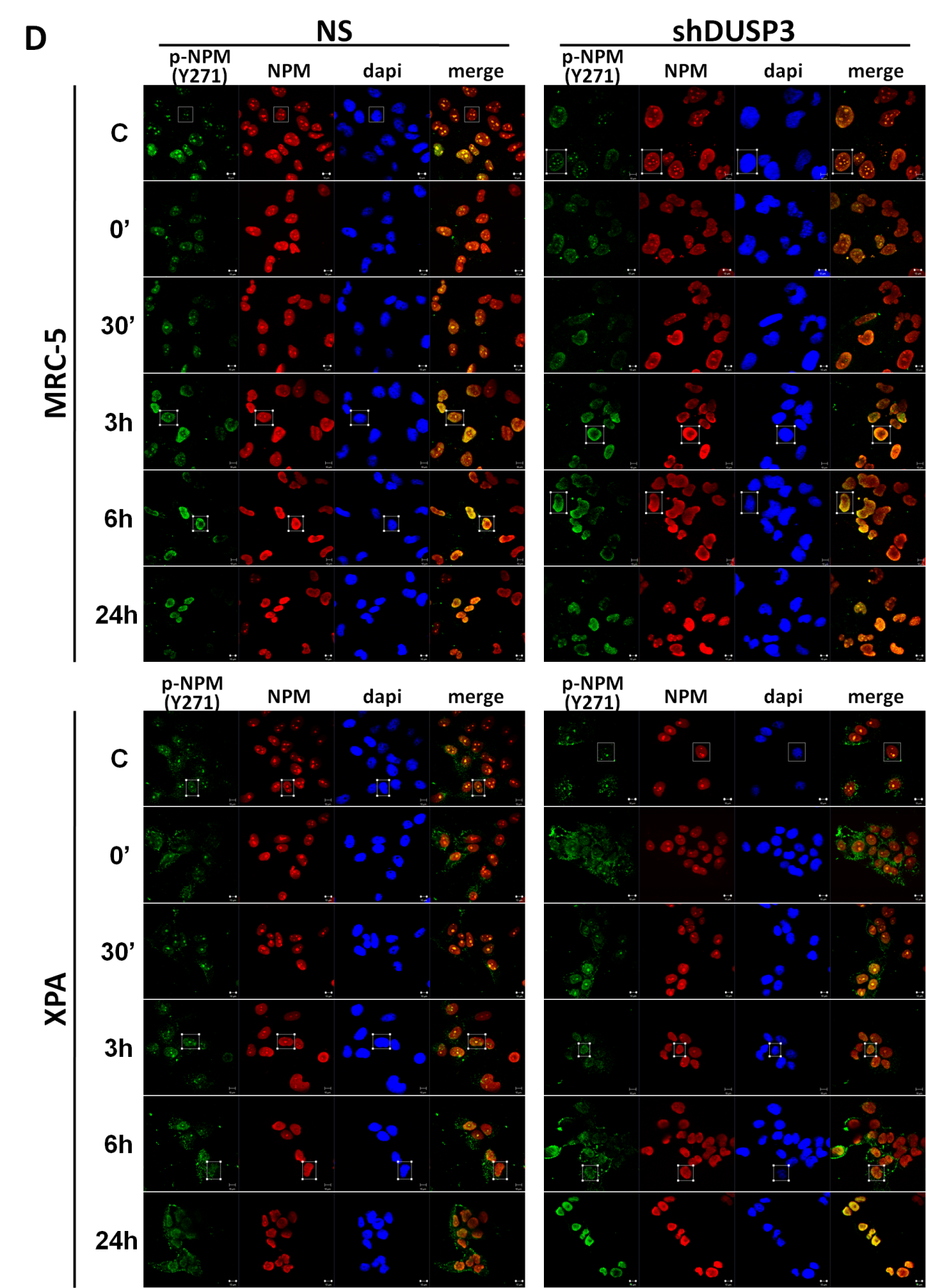


**Fig. S3. Immunolocalization of the NPM phosphorylated at the four tyrosines.** MRC-5 and XPA cells (NS and shDUSP3) were exposed to 18 J/m^2^ UVC and collected for immunofluorescences after 0 min, 30 min, 3 hours, 6 hours, and 24 hours. Images are qualitative and representative of three independent experiments in triplicate totalizing at least 100 nuclei. The white squares represent the cell nucleus shown in Fig. 3 and white bar scale indicates 10 µm length at 63x magnification. The yellow color at the merge micrographs represents the staining of total NPM protein plus NPM phosphorylated in each tyrosine: (**A**) p-NPM(Y17); (**B**) p-NPM(Y29); (**C**) p-NPM(Y67); (**D**) p-NPM(Y271). The Y29, Y67 and Y271 residues of NPM seem to be most relevant since they remain phosphorylated during all the kinetic of nucleolus-nucleoplasm translocation of NPM. Most importantly is that phospho-Y- NPM translocation occurs as early as 3 hours after UVC radiation in both shDUSP3 cells.

**Figure S4**

**
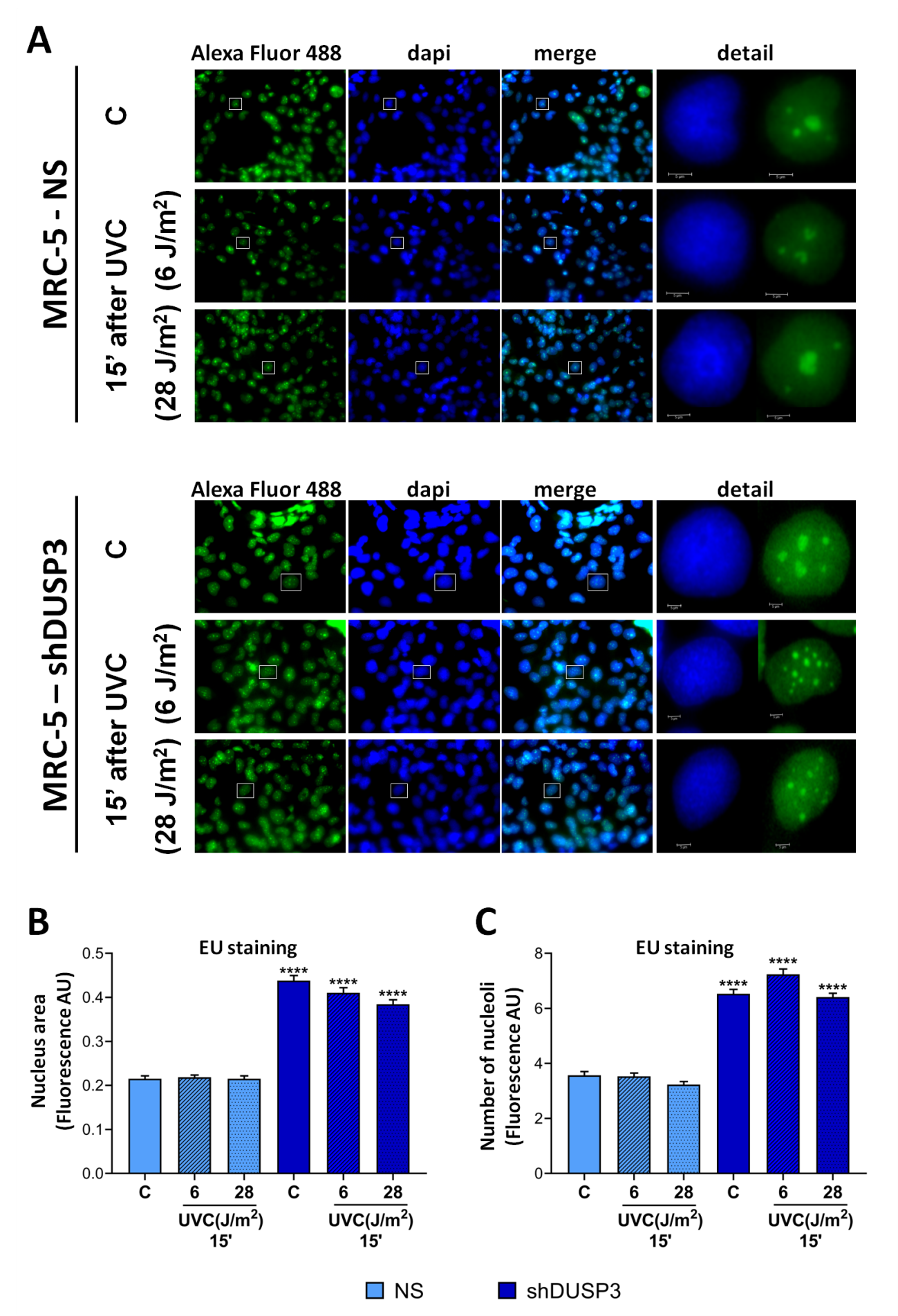
**

**Fig. S4. DUSP3 knockdown affects global RNA transcription rate**. (**A**) MRC-5 NS or shDUSP3 cells were incubated with the probe 5-ethynyl uridine (EU) conjugated with Alexa Fluor 488, exposed to two different doses of UVC radiation and 15 min latter submitted to staining assay using the Click-iT Nascent RNA capture kit (Thermo Fischer Scientific). The nucleoli of 100 individual cells were counted. Images were obtained in a Leica DMi8 fluorescence microscope, at 40x magnification and a total fluorescence intensity of 100 individual nuclei measured with the ImageJ Software. Representative images represent the quantification graphs from three independent experiments shown in Fig. 3H. Higher basal levels of global RNA transcription were observed in MRC-5 cells under DUSP3 silencing. A representative cell nucleus inside the white square is showed zoomed and white bar scale indicates 5 µm length. (**B**) The nucleus area was measured and plotted as arbitrary units (AU) from micrographs shown in A. MRC-5 shDUSP3 cells have a larger nucleus than the control NS cells. (**C**) Areas of high fluorescence intensity, usually assumed as areas of high ribosomal DNA transcription, were assumed as nucleolar regions for indirect measurements of nucleolar morphology (size and number). These results corroborate that shown in Fig. 3F where DUSP3 knockdown cells showed greater number of nucleoli than NS cells. Anova: ****: p<0.0001.

**Figure S5**


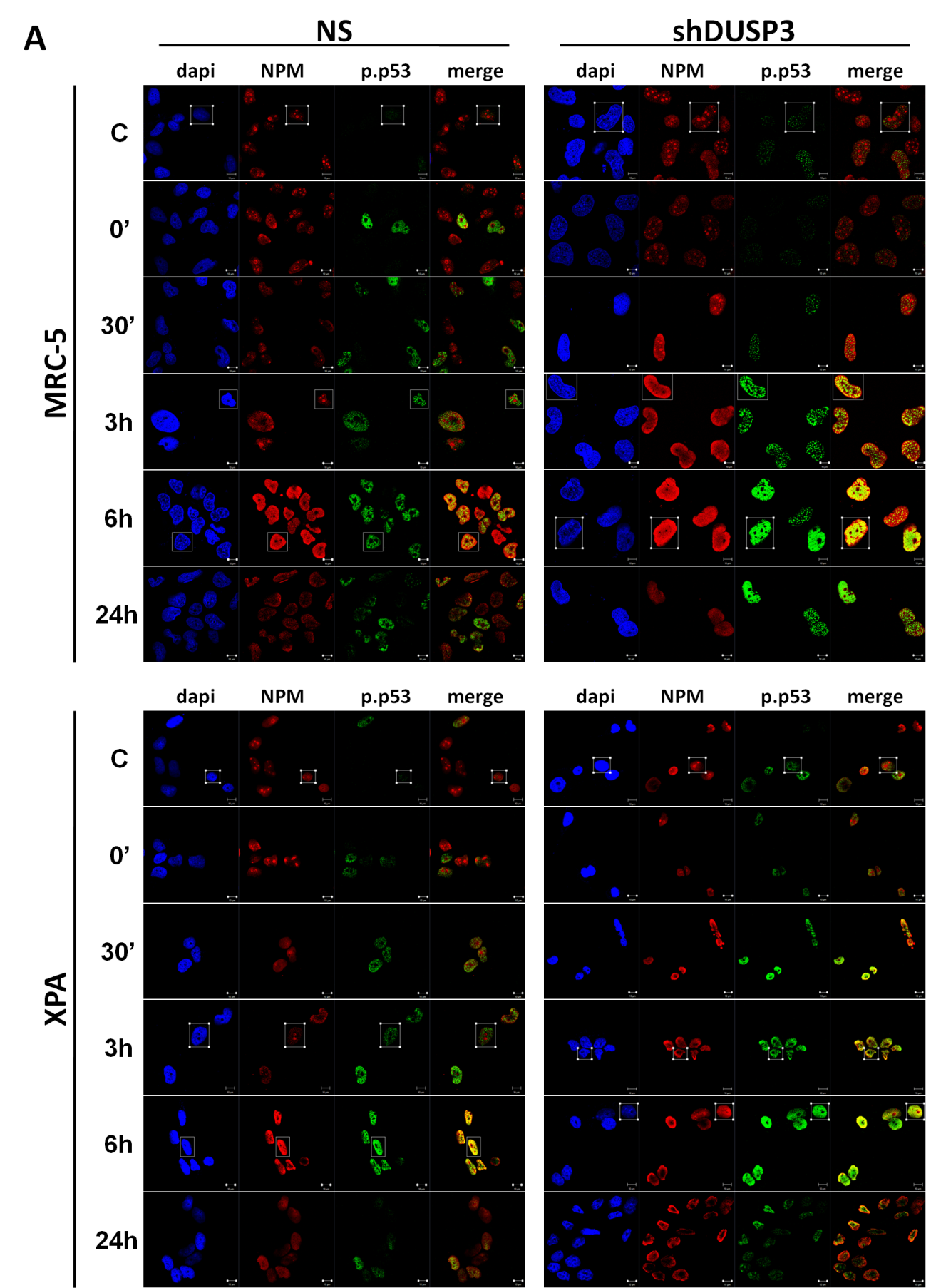


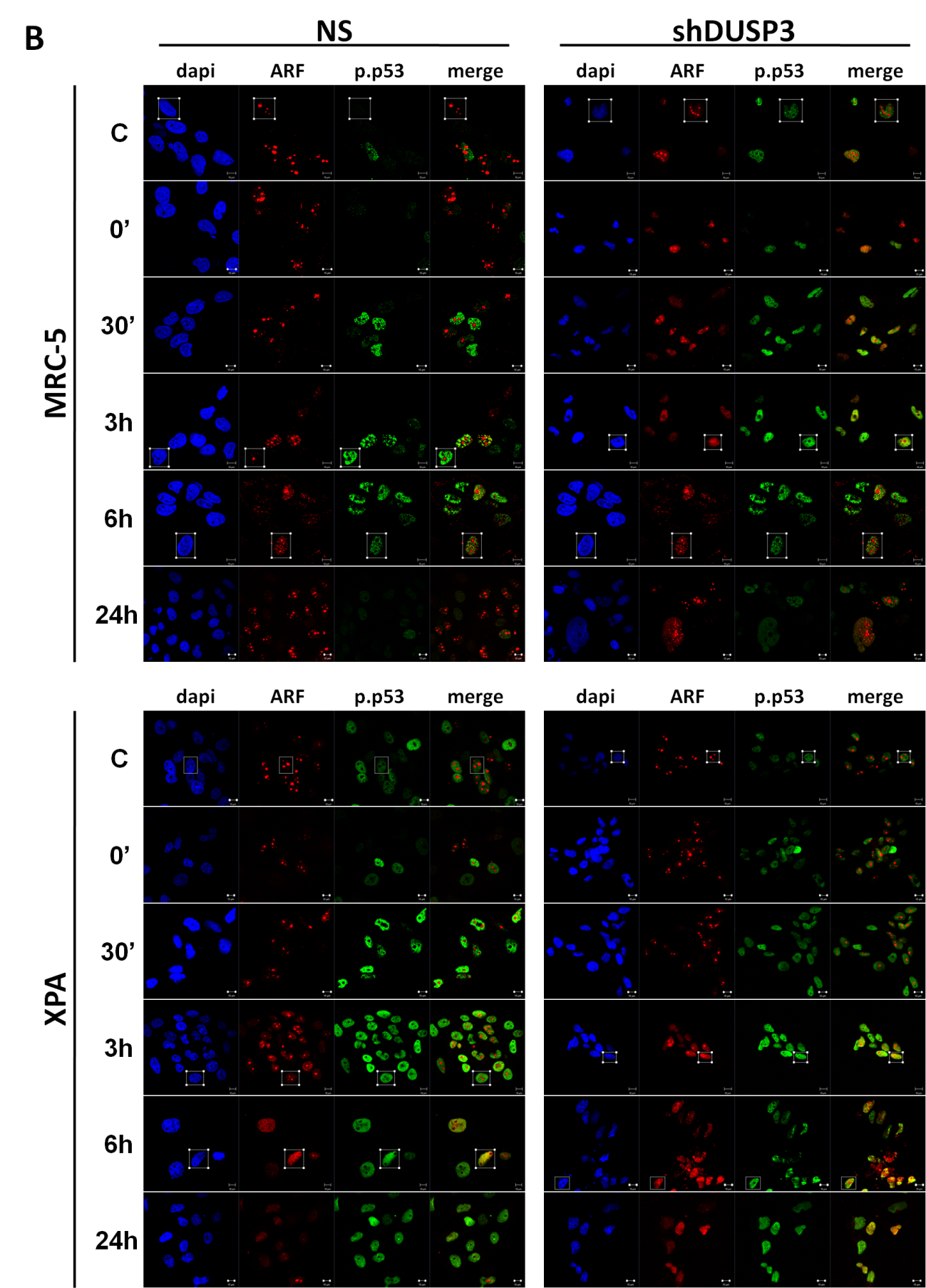


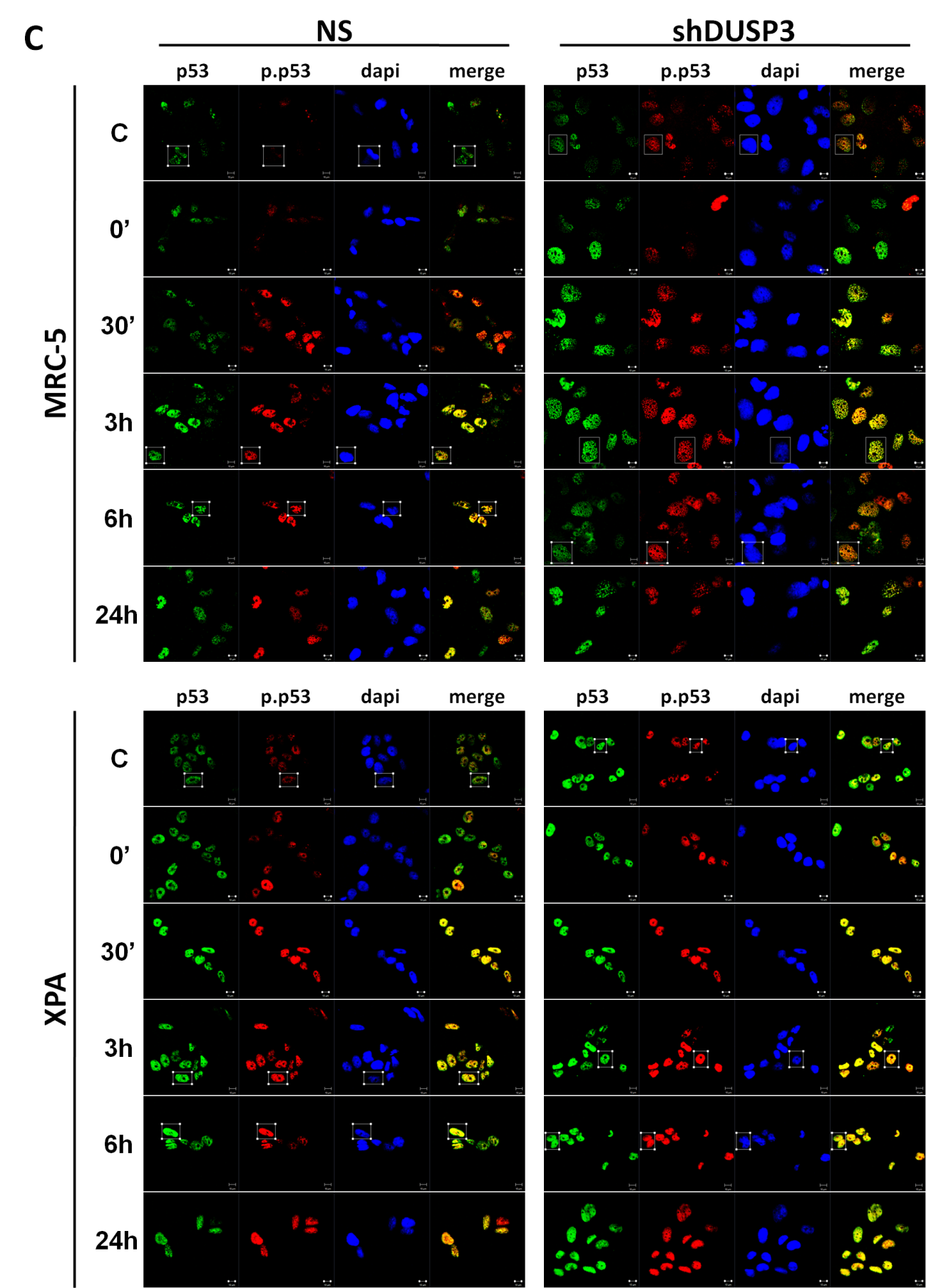


**Fig. S5. The nucleolus-nucleoplasm translocation of NPM and ARF proteins after UV radiation affected p53(Ser15) phosphorylation in a DUSP3-dependent manner.** MRC-5 and XPA cells (NS and shDUSP3) were exposed to 18 J/m^2^ UVC and collected for immunofluorescence after 0 min, 30 min, 3 hours, 6 hours, and 24 hours and incubated as indicated using double labeling. The white squares indicate the representative cell nucleus showed in the Fig. 4 and white bar scale indicates 10 µm length at 63x magnification. The yellow color at the merge micrographs represents the colocalization intensity between two labeled proteins. **(A, B)** NPM and ARF, respectively, translocated as early as 3 hours from nucleolus to nucleoplasm and colocalized with phosphorylated p53(Ser15) (p-p53) markedly in shDUSP3 cells. **(C)** In both DUSP3 silenced cells, p-p53 is already observed in basal conditions (control C and 0 min after UVC) as also visualized in the Fig. 2. The levels of p-p53 reached a maximum in 3 hours in shDUSP3 cells, whereas only in 6 hours after UVC exposure in NS cells.

**Figure S6**


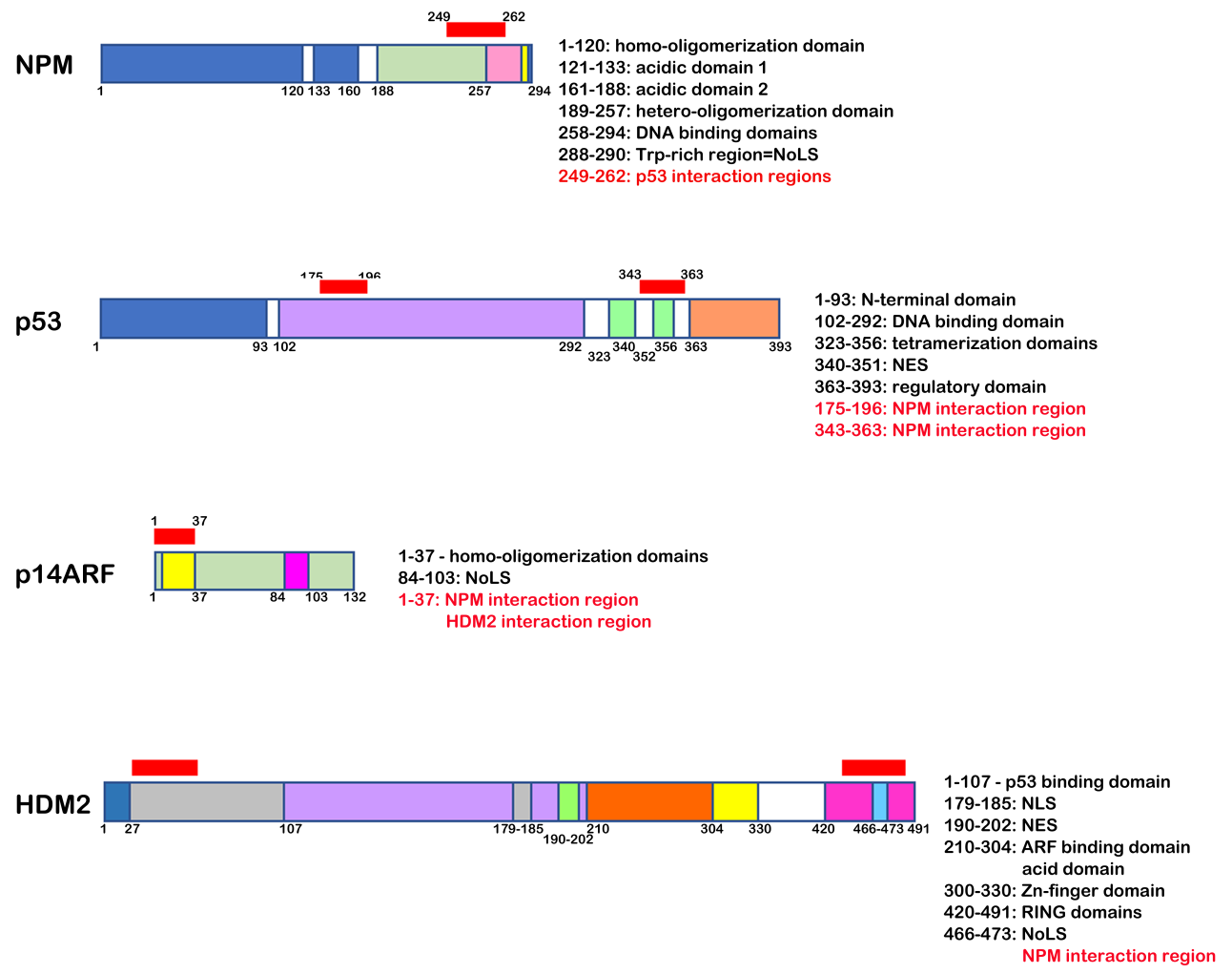


**Fig S6. Schematic illustration of the main regions involved in the binary interactions between NPM, p53, ARF and HDM2 proteins.** The extended primary structures of each one of four proteins comprise the more relevant domains, motifs, and amino acid sequences for their main biological functions, and they also highlight in the thick red traces the recognized and experimentally demonstrated interacting regions between these four proteins.

**Figure S7**


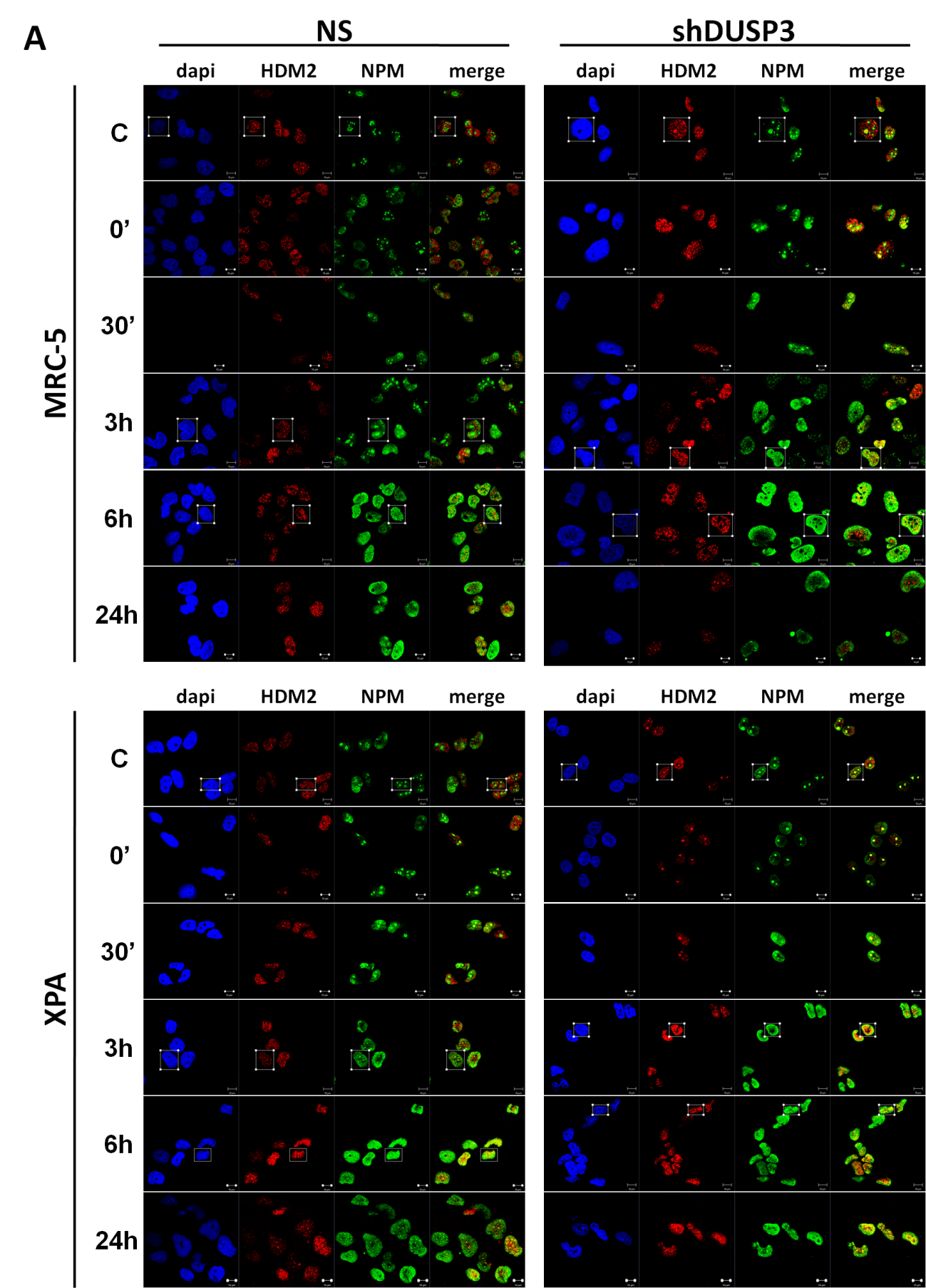


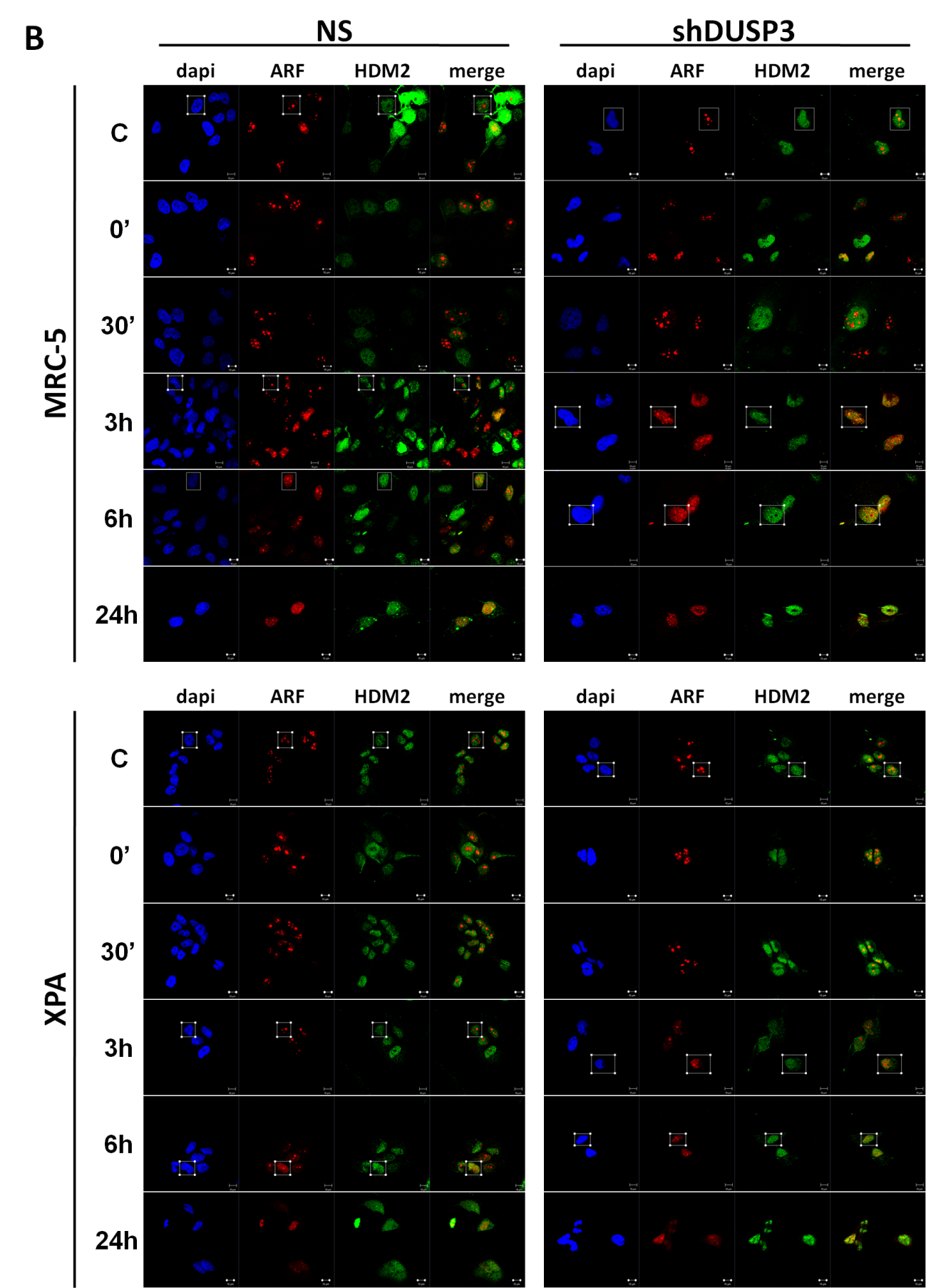


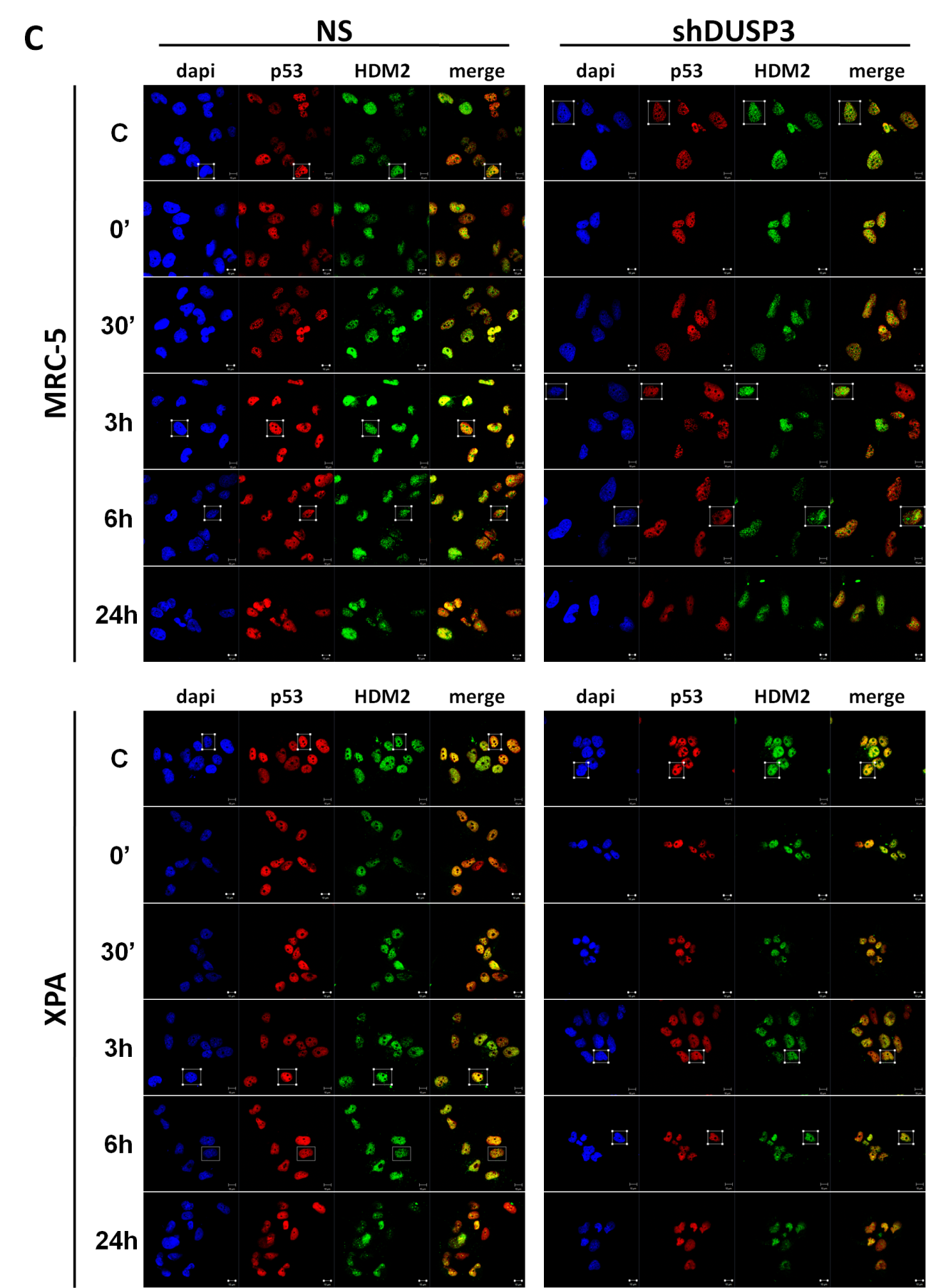


**Fig. S7. DUSP3 stabilizes p53 through the modulation of NPM/ARF-HDM2 interaction.** MRC-5 and XPA cells (NS and shDUSP3) were exposed to 18 J/m^2^ UVC and collected for immunofluorescence after 0 min, 30 min, 3 hours, 6 hours, and 24 hours and incubated as indicated using double labeling. The white squares indicate the representative cell nucleus showed in the Fig. 5 and white bar scale indicates 10 µm length at 63x magnification. The yellow color in the merge micrographs represents the colocalization intensity between two labeled proteins. **(A, B)** NPM and ARF, respectively, translocated as early as 3 hours after UVC to nucleoplasm and strongly colocalized with HDM2 especially the shDUSP3 cells. **(C)** Interestingly the opposite was found in the colocalization between p53 and HDM2, which is disrupted earlier in shDUSP3 cells (3 hours after UVC) in relation to NS cells. HDM2 is seen in the nucleus of shDUSP3 cells even in the basal conditions (control non-irradiated cells), which may influence p53 stabilization and phosphorylation in the nucleoplasm after UVC exposure.

**Figure S8**

**
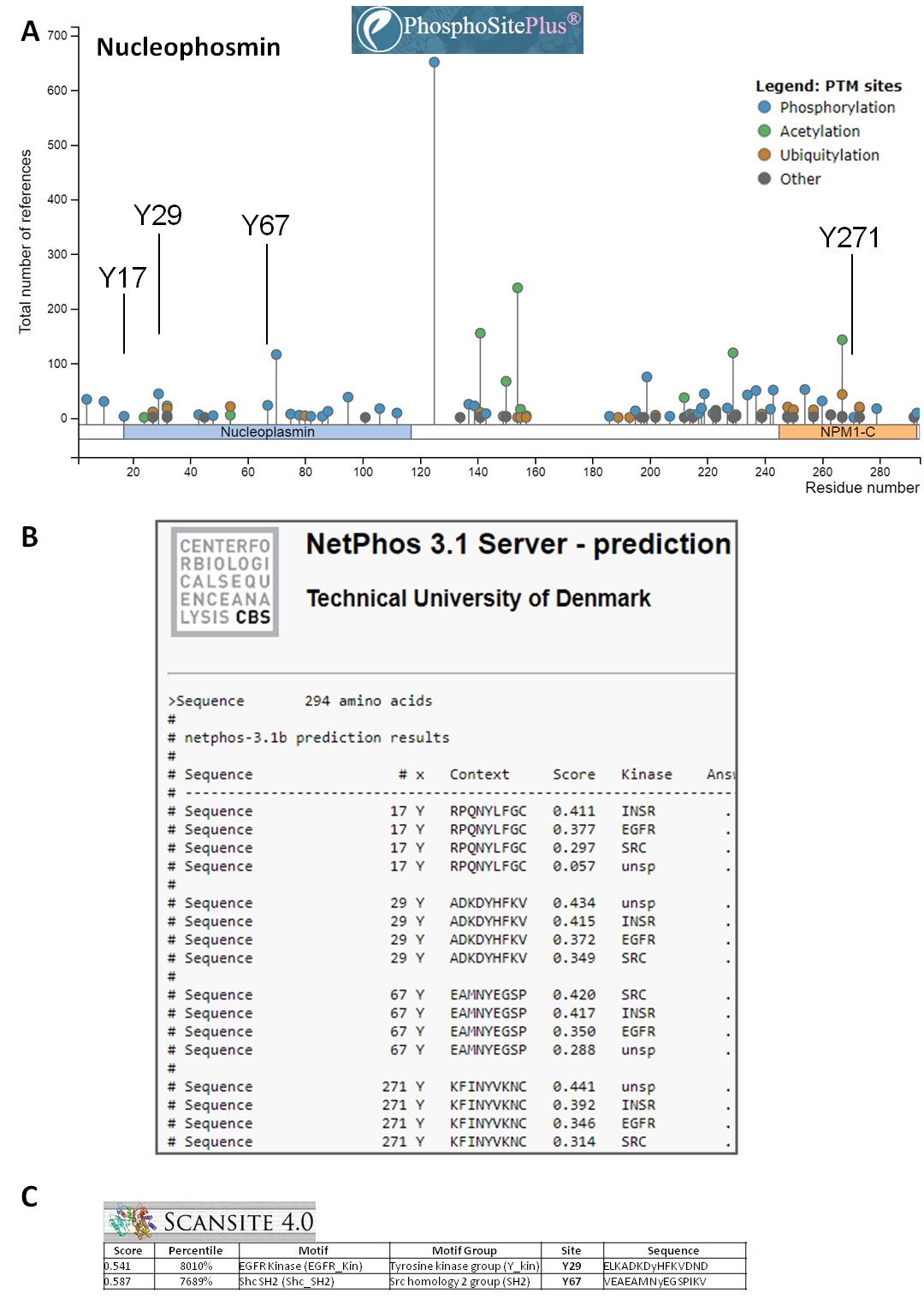
**

**Fig S8. Analyses of phosphorylation prediction of phosphosites on NPM suggest high probability of existing phosphorylated tyrosines.** Three different bioinformatics prediction tools, using different algorithms and based on experimental phosphopeptide databases - **(A)** PhosphoSitePlus, **(B)** NetPhos and **(C)** Scansite - confirmed NPM has remarkably high probability of being phosphorylated on its four tyrosine residues (Y17, Y29, Y67 and Y271). However, NPM tertiary and quaternary structures (shown in Fig. 2A) suggest the last three tyrosines are prone to post-translation modifications mainly due to their more exposed positions.

**TABLES**

**Supplementary Table 1.** Surface Plasmon Resonance Analysis: KD values for the bimolecular interaction between DUSP3 (WT or C124S) and NPM full length, NPM_9-122_ or ERK1, as well as for DUSP3-WT and the phospho-Y-decapeptides of NPM.

| **Analyte**  **Ligand** | **ERK1** | **NPM**  **full length** | **NPM**  **9-122** | **pNPM-Y17** | **pNPM-Y29** | **pNPM-Y67** | **pNPM-Y271** |
| --- | --- | --- | --- | --- | --- | --- | --- |
| **DUSP3-WT** | 178 x10^-9^  ± 27 x10^-9^ | 17.2 x10^-9^  ± 3.7 x10^-9^ | 294 x10^-9^  ± 13 x10^-9^ | 1.62 x10^-6^  ± 0.17 x10^-6^ | 2.34 x10^-6^  ± 0.16 x10^-6^ | 2.01 x10^-6^  ± 0.27 x10^-6^ | 2.07 x10^-6^  ± 0.1 x10^-6^ |
| **DUSP3-C124S** | 130 x10^-9^  ± 29 x10^-9^ | 11.5 x10^-9^  ± 2.6 x10^-9^ | 393 x10^-9^  ± 56 x10^-9^ | - | - | - | - |

**Supplementary Table 2.** KD values measured by SPR technique used as reference.

| **Ligand** | **Analyte** | **KD (M)** |
| --- | --- | --- |
| VEGF (*1*) | rhVEGFR-1 | 2.85 x10^-10^ |
| TNFR (*2*) | TRADD | 2.5 x10^-8^ |
| TNFR (*2*) | RIP1 | 5.43 x10^-8^ |
| Rpn10 (26S proteasome regulatory subunit) (*3*) | pyruvate kinase | 2.8 x10^-5^ |
| H2B (*3*) | Rpn10 | 3.2 x10^-9^ |
| proMMP-2:TIMP-2 complex (*4*) | megalin/LRP-2 | 18.6 x10^-9^ |
| Tyrosinase (*5*) | hydroquinone | 2.43 x10^-7^ |
| Caveolin-1 (*6*) | PEDF (Pigment epithelium-derived factor) | 7.36 x10^-7^ |
| beta-amyloid peptide(*7*) | peroxiredoxin | 8.36 х10^-8^ |
| beta-amyloid peptide (*7*) | alpha-enolase | 1.97 х10^-6^ |
| PDGFR-β extracellular domain (*8*) | destruxin A5 natural cyclopeptide | 8.02 х10^-6^ |
| GRP78, a specific cancer cell-surface marker (*9*) | L-peptide RPLLPY | 1.10 x10^-6^ |

**Supplementary Table 3.** Technical specifications of the antibodies used for double staining assays in the confocal microscopy analysis.

| **Antibody**  **1** | **Manufacturer**  **Cat #** | **Dilution**  **Used** | **Secondary**  **Antibody** | **Antibody 2**  **2** | **Manufacturer**  **Cat #** | **Dilution**  **Used** | **Secondary**  **Antibody** |
| --- | --- | --- | --- | --- | --- | --- | --- |
| **p-NPM (Y17, Y29, Y67, Y271)** | Celula B | 1:50 | chicken anti-rabbit Alexa 488 | **NPM** | Sigma-Aldrich  cat B0556 | 1:500 | goat anti-mouse Alexa 555 |
| **p-p53(S15)** | Cell Signaling  cat 9284 | 1:300 | chicken anti-rabbit Alexa 488 | **NPM** | Sigma-Aldrich  cat B0556 | 1:500 | goat anti-mouse Alexa 555 |
| **DUSP3** | BD  cat 610547 | 1:50 | chicken anti-rabbit Alexa 488 | **NPM** | Sigma-Aldrich  cat B0556 | 1:500 | goat anti-mouse Alexa 555 |
| **p53** | Santa Cruz  cat sc-56180 | 1:300 | chicken anti-rabbit Alexa 488 | **p-p53 (S15)** | Cell Signaling  cat 9284 | 1:300 | goat anti-mouse Alexa 555 |
| **HDM2** | Cell Signaling  cat 86934 | 1:200 | chicken anti-rabbit Alexa 488 | **p53** | Santa Cruz  cat sc-56180 | 1:300 | goat anti-mouse Alexa 555 |
| **NPM** | Sigma-Aldrich  cat B0556 | 1:500 | chicken anti-mouse Alexa 488 | **HDM2** | Cell Signaling  cat 86934 | 1:200 | goat anti-rabbit Alexa 555 |
| **HDM2** | Cell Signaling  cat 86934 | 1:200 | chicken anti-rabbit Alexa 488 | **ARF** | Santa Cruz  cat sc-73434 | 1:100 | goat anti-mouse Alexa 555 |
| **p-p53 (S15)** | Cell Signaling  cat 9284 | 1:300 | chicken anti-rabbit Alexa 488 | **ARF** | Santa Cruz  cat sc-73434 | 1:100 | goat anti-mouse Alexa 555 |
| **NAT10** | Sigma-Aldrich  cat SAB2700591 | 1:200 | chicken anti-rabbit Alexa 488 | **HDM2** | Santa Cruz  cat sc-965 | 1:100 | goat anti-mouse Alexa 555 |

**SUPPLEMENTARY REFERENCES**

1. J. Wang, M. Shi, Y. Xi, L. Gao, G. Zhang, Y. Shao, H. Chen, X. Hu, Recombinant human vascular endothelial growth factor receptor 1 effectively inhibits angiogenesis in vivo. *Mol. Med. Rep.* **11**, 3432–8 (2015).

2. Y. H. Park, M. S. Jeong, S. B. Jang, Death domain complex of the TNFR-1, TRADD, and RIP1 proteins for death-inducing signaling. *Biochem. Biophys. Res. Commun.* **443**, 1155–1161 (2014).

3. O. A. Buneeva, O. V Gnedenko, A. T. Kopylov, M. V Medvedeva, V. G. Zgoda, A. S. Ivanov, A. E. Medvedev, Quantitative Affinity Interaction of Ubiquitinated and Non-ubiquitinated Proteins with Proteasome Subunit Rpn10. *Biochemistry. (Mosc).* **82**, 1042–1047 (2017).

4. M. Johanns, P. Lemoine, V. Janssens, G. Grieco, S. K. Moestrup, R. Nielsen, E. I. Christensen, P. J. Courtoy, H. Emonard, E. Marbaix, P. Henriet, Cellular uptake of proMMP-2:TIMP-2 complexes by the endocytic receptor megalin/LRP-2. *Sci. Rep.* **7**, 4328 (2017).

5. S. Patil, S. Sistla, J. Jadhav, Interaction of small molecules with human tyrosinase: A surface plasmon resonance and molecular docking study. *Int. J. Biol. Macromol.* **92**, 1123–1129 (2016).

6. T. Matsui, Y. Higashimoto, J. Taira, S. I. Yamagishi, Pigment epithelium-derived factor (PEDF) binds to caveolin-1 and inhibits the pro-inflammatory effects of caveolin-1 in endothelial cells. *Biochem. Biophys. Res. Commun.* **441**, 405–410 (2013).

7. O. A. Buneeva, O. V Gnedenko, M. V Medvedeva, A. S. Ivanov, A. E. Medvedev, [The effect of neuroprotector isatin on binding of some model proteins with beta-amyloid peptide: a biosensor study]. *Biomed. Khim.* **62**, 720–724 (2016).

8. X. Wang, X. Wu, A. Zhang, S. Wang, C. Hu, W. Chen, Y. Shen, R. Tan, Y. Sun, Q. Xu, Targeting the PDGF-B/PDGFR-β Interface with Destruxin A5 to Selectively Block PDGF-BB/PDGFR-ββ Signaling and Attenuate Liver Fibrosis. *EBioMedicine*. **7**, 146–56 (2016).

9. S.-H. Wang, A. C.-L. Lee, I.-J. Chen, N.-C. Chang, H.-C. Wu, H.-M. Yu, Y.-J. Chang, T.-W. Lee, J.-C. Yu, A. L. Yu, J. Yu, Structure-based optimization of GRP78-binding peptides that enhances efficacy in cancer imaging and therapy. *Biomaterials*. **94**, 31–44 (2016).
